# Plasma Metabolite Markers of Parkinson’s Disease and Atypical Parkinsonism

**DOI:** 10.3390/metabo11120860

**Published:** 2021-12-09

**Authors:** Meerakhan Pathan, Junfang Wu, Hans-Åke Lakso, Lars Forsgren, Anders Öhman

**Affiliations:** 1 Department of Integrative Medical Biology, Umeå University, SE-901 87 Umeå, Sweden; meerakhanpathan82@gmail.com (M.P.); junfang.wu@hotmail.com (J.W.); 2 Division of Cardiology, Department of Internal Medicine, Tongji Hospital, Tongji Medical College, Huazhong University of Science and Technology, Wuhan 430030, China; 3 Department of Medical Biosciences, Umeå University, SE-901 85 Umeå, Sweden; HansAke.Lakso@regionvasterbotten.se; 4 Department of Clinical Sciences, Neurosciences, Umeå University, SE-901 85 Umeå, Sweden; lars.forsgren@umu.se

**Keywords:** Parkinson’s disease, multiple system atrophy, progressive supranuclear palsy, nuclear magnetic resonance, mass spectrometry, metabolomics, atypical Parkinsonism, plasma, biomarker

## Abstract

Differentiating between Parkinson’s disease (PD) and the atypical Parkinsonian disorders of multiple system atrophy (MSA) and progressive supranuclear palsy (PSP) is difficult clinically due to overlapping symptomatology, especially at early disease stages. Consequently, there is a need to identify metabolic markers for these diseases and to develop them into viable biomarkers. In the present investigation, solution nuclear magnetic resonance and mass spectrometry metabolomics were used to quantitatively characterize the plasma metabolomes (a total of 167 metabolites) of a cohort of 94 individuals comprising 34 PD, 12 MSA, and 17 PSP patients, as well as 31 control subjects. The distinct and statistically significant differences observed in the metabolite concentrations of the different disease and control groups enabled the identification of potential plasma metabolite markers of each disorder and enabled the differentiation between the disorders. These group-specific differences further implicate disturbances in specific metabolic pathways. The two metabolites, formic acid and succinate, were altered similarly in all three disease groups when compared to the control group, where a reduced level of formic acid suggested an effect on pyruvate metabolism, methane metabolism, and/or the kynurenine pathway, and an increased succinate level suggested an effect on the citric acid cycle and mitochondrial dysfunction.

## 1. Introduction

Parkinson’s disease (PD) is a common neurodegenerative disorder characterized by loss of dopaminergic neurons in the substantia nigra pars compacta and intraneuronal α-synuclein-rich protein aggregates (Lewy bodies and Lewy neurites) [1,2]. Diagnosis of PD is based on the presence of bradykinesia and at least one other symptom of resting tremor, rigidity, and postural instability [3]. Additional motor and non-motor symptoms are frequently observed [2]. However, differentiating PD from the atypical Parkinsonian disorders of multiple system atrophy (MSA) and progressive supranuclear palsy (PSP) can be challenging due to overlapping symptomatology, especially at early stages of the disease [4,5,6]. MSA is similar to PD an α-synucleinopathy, but with deposits primarily in the glial cell population, whereas PSP instead is a tauopathy displaying neurofibrillary tangles of the tau protein. Both MSA and PSP are further divided into subgroups where those with a Parkinsonian symptomatology are denoted MSA-P and PSP-P, respectively.

Numerous approaches have been applied to differentiate between these disorders and to obtain a correct diagnosis, among them clinical decision trees [7], several types of imaging techniques [8,9,10], and the use of potential biomarkers primarily in cerebrospinal fluid (CSF). Although partly successful in clarifying the diagnostic uncertainties, there is currently no widely accepted approach for use in the routine diagnostic investigation of Parkinsonian disorders. Therefore, it is desirable to develop a simple, timesaving, inexpensive, and non-invasive clinical test that can assist in the early diagnosis of Parkinsonian disorders.

Many of the potential biomarkers previously tested are single proteins detected in CSF, such as Parkinson disease protein 7, α-synuclein, total tau protein, soluble amyloid precursor protein α, and neurofilament light chain protein or a combination of these proteins [11,12,13,14,15,16], but CSF catecholamines are also used [17]. Although these markers in part are successful, there is a need to identify viable biomarkers in a more easily accessible biofluid. Consequently, numerous omic-techniques have been employed, and in the present paper we have used metabolomics. Metabolomics/metabonomics is the quantitative analysis of metabolites in a living system and facilitates the detection of the metabolic response to a pathophysiological state [18]. Nuclear magnetic resonance (NMR) spectroscopy and mass spectrometry (MS) methods are the most frequently used techniques, where NMR is an inexpensive, robust, and inherently quantitative, but less sensitive, technique. MS is a more sensitive technique, and the introduction of targeted analysis kits, such as the Absolute*IDQ^®^* p180 kit (Biocrates Life Science, Innsbruck, Austria), provides an accessible, reproducible, and quantitative analysis platform. Numerous metabolomic studies of PD patients have shown significant alterations of the metabolome in CSF [19,20] as well as in the more easily accessible serum, plasma, and urine biofluids [21,22,23,24,25,26,27,28,29]. One NMR study investigated the serum metabolome of the three Parkinsonian disorders (PD, MSA, and PSP) [30], and an MS study analyzed the MSA and PSP metabolomes in plasma [31], both providing important clues to the metabolic changes that occur in these Parkinsonian disorders.

In the present study, solution NMR spectroscopy and MS spectrometry metabolomics were used together for the first time to quantitatively characterize the plasma metabolome from individuals in four age and gender-matched groups, comprising PD, MSA, and PSP patients and a control group. Plasma is an easily accessible biofluid that would be highly advantageous to use clinically for diagnostic purposes. The combined analysis using both NMR and MS data revealed significant differences in the metabolite concentrations between the different groups and identified potential plasma metabolite markers for each disorder.

## 2. Results

The use of both NMR and MS-based techniques for the quantitative analysis of metabolites resulted in two datasets that were initially analyzed separately and then merged and analyzed together. The results from the separate analyses are briefly presented here and thoroughly described in the Supplement. The results obtained from the merged data set are presented in detail below.

### 2.1. NMR-Based Analysis

The recorded ^1^H-NMR spectra of the 94 filtered plasma samples were analyzed in the Chenomx software, and a total of 49 metabolites were identified, and of these 34 were quantified in all samples. A list of the quantified metabolites and their concentration averages and standard deviations for the respective disease and control groups is found in Supplementary Table S1. All of the metabolites had concentrations in the same ranges as previously observed [32].

An initial multivariate statistical analysis of the NMR data using principal component analysis (PCA) revealed no significant outliers. However, one PD patient and two PSP patients were under levodopa treatment, and to avoid influencing the statistical analysis they were excluded. To investigate the possibility for discriminating between PD, MSA, PSP, and control groups, the metabolite data for the different groups were compared pairwise using orthogonal projections of latent structure-discriminant analysis (OPLS-DA). The results based on the NMR data alone are thoroughly presented in the Supplement and include an overview of the statistical parameters (Table S3), the cross-validated score plots, the most important discriminatory metabolites, and the ROC analysis (Figures S1 and S2) for each pairwise comparison. The fold changes (*fc*), *p*-values (from the univariate analysis), and weight values (w*, from the multivariate analysis) for all metabolites are listed in Table S4. Based on the initial OPLS-DA modeling involving the PSP group, it was apparent that one subject was a clear outlier in the PSP group and clearly disturbed the modeling. This patient was the only PSP patient displaying pure akinesia with gait freezing (PSP-pagf), and consequently this patient was removed from the modeling.

### 2.2. MS-Based Analysis

The 94 unfiltered plasma samples were analyzed using the Biocrates Absolute*IDQ^®^* p180 MS kit together with the Analyst and the MetIDQ software packages. This kit enables quantification of at most 188 metabolites; after performing the standardized validation procedure within MetIDQ, it resulted in the quantification of 133 metabolites. The quantified metabolites and their concentration averages and standard deviations for the respective disease and control groups are listed in Supplementary Table S2. Ten of the control samples lacked data for taurine due to a technical issue, and therefore taurine was excluded as a variable when comparisons with the control group were performed. An initial analysis of the MS data using PCA revealed no outliers. Similar to the NMR analysis, three subjects on levodopa and one PSP-pagf patient were not included in the OPLS-DA of the MS data. The results for each pair-wise comparison of groups are presented in the Supplement and include an overview of the statistical parameters (Table S3), the cross-validated score plots, the most important discriminatory metabolites, and the ROC analysis (Figures S3 and S4). The fold changes, *p*-values (from the univariate analysis), and w* (from the multivariate analysis) for all metabolites are listed in Table S5.

### 2.3. Analysis of the Merged NMR- and MS-Data

The NMR and MS data from the 94 subjects were merged, and the disease groups and control group were analyzed pairwise using OPLS-DA. In total 167 metabolites (34 from the NMR data and 133 from the MS data, where 17 metabolites overlapped) were utilized, and due to the reasons described above three patients under levodopa treatment and the single PSP-pagf patient, as well as the MS-detected metabolite taurine, were excluded from the analysis. The parameters used in the OPLS-DA modeling and the CV-ANOVAs and AUCs of each pair-wise comparison are listed in Table 1. The results obtained from the statistical analysis are described in detail below.

**Figure 1:**
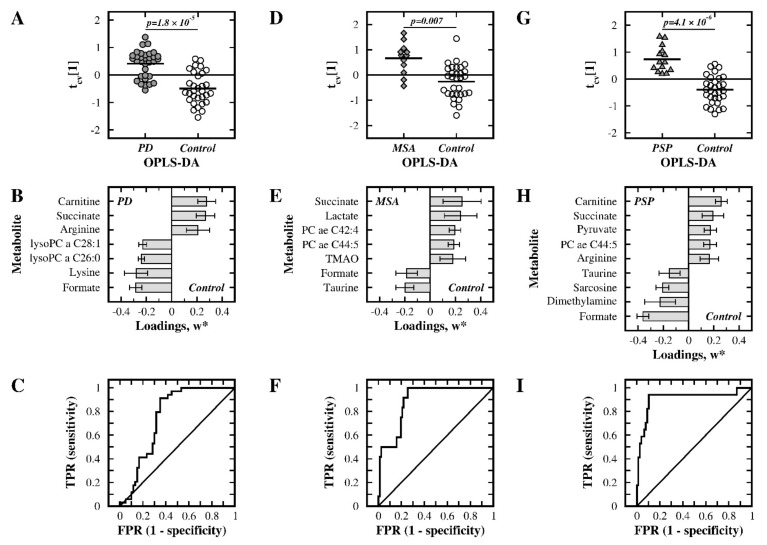
Multivariate statistical comparisons between the patient groups and control group using the merged NMR and MS data. (**A**–**C**) PD versus controls, (**D**–**F**) MSA versus controls, and (**G**–**I**) PSP versus controls. Cross-validated OPLS-DA score plots of the first component, t_cv_ [1], are shown in panels (**A**,**D**,**G**). The most important metabolites in each model, as judged from their w*, are displayed in panels (**B**,**E**,**H**). ROC analyses for discriminating between groups are presented in panels (**C**,**F**,**I**). TPR: true positive rate; FPR: false positive rate; TMAO: trimethylamine N-oxide; lysoPC: lysophosphatidylcholine; PC: phosphatidylcholine.

For the PD and control groups, the OPLS-DA clearly separated the two groups and provided a statistically significant model (Q^2^ = 0.417, *p* = 1.8 × 10^−5^, Table 1). The resulting cross-validated score plot is shown in Figure 1A, and discriminatory metabolites (with |w*| > 0.2) are listed in Figure 1B. The accompanying ROC analysis is shown in Figure 1C and illustrates a fair discriminatory ability with an AUC of 0.76. The univariate analysis using Student’s *t*-test showed that all of the identified metabolites had a *p*-value below 0.05 (Tables S4 and S5).

In the OPLS-DA of the MSA and control groups, a statistically significant separation of groups (Q^2^ = 0.302, *p* = 0.007, Table 1) was observed, as shown in the cross-validated score plot in Figure 1D. The most important metabolites (with |w*| > 0.175) for discrimination are shown in Figure 1E. The ROC analysis, shown in Figure 1F, indicates a good discriminatory ability with an AUC of 0.89. Six of these seven metabolites had a *p*-value below 0.05 in the univariate analysis (Tables S4 and S5). 

When PSP patients were compared with controls, a statistically significant OPLS-DA model (Q^2^ = 0.524, *p* = 4.1 × 10^−6^, Table 1) was obtained. The cross-validated score plot of this model is shown in Figure 1G, and metabolites with |w*| above 0.15 are shown in Figure 1H. The ROC analysis had very good discriminating ability with an AUC of 0.91, as shown in Figure 1I. All of the identified metabolites were also of importance in the univariate analysis (*p* < 0.05; Tables S4 and S5).

**Figure 2:**
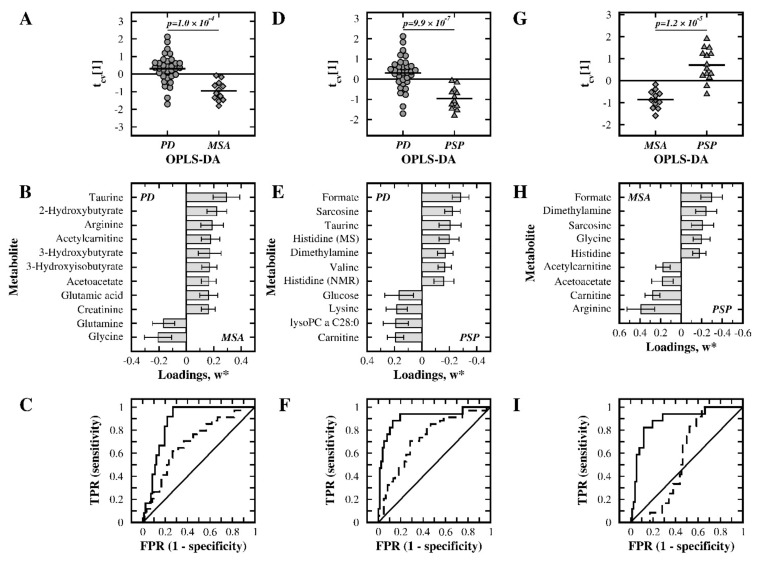
Multivariate statistical comparisons between the patient groups using the merged NMR and MS data. (**A**–**C**) PD versus MSA, (**D**–**F**) PD versus PSP, and (**G**–**I**) MSA versus PSP. Cross-validated OPLS-DA score plots of the first component, t_cv_ [1], are shown in panels (**A**,**D**,**G**). The most important metabolites in each model, as judged from their w*, are displayed in panels (**B**,**E**,**H**). ROC analyses for discriminating between groups are presented in panels (**C**) (PD group: solid line), (**F**) (PD group: solid line) and (**I**) (MSA group: solid line). TPR: true positive rate; FPR: false positive rate; TMAO: trimethylamine N-oxide; lysoPC: lysophosphatidylcholine; PC: phosphatidylcholine.

Furthermore, pairwise OPLS-DA was used to discriminate the different disease groups from each other. In the comparison between PD and MSA, a significant model was not obtained (Q^2^ = 0.091, *p* = 0.420). By selecting the most important metabolites (with |w*| > 0.175) from that model, a second round of modeling gave a statistically significant model (Q^2^ = 0.355, *p* = 1.0 × 10^−4^; Table 1). Figure 2A,B show the cross-validated score plot of this model and the weight values of the 11 retained metabolites, respectively. The ROC analysis showed a fair discriminatory ability with an AUC of 0.70 for the PD group and a good discriminatory ability with an AUC of 0.87 for the MSA group, see Figure 2C. Seven out of these eleven metabolites had a *p*-value below 0.05 in Student’s *t*-test (Tables S4 and S5).

In the OPLS-DA of the PD and PSP groups, the obtained model lacked statistical significance (Q^2^ = 0.140, *p* = 0.165). Metabolites with |w*| > 0.15 were selected, and a valid model was obtained (Q^2^ = 0.467, *p* = 9.9 × 10^–7^; Table 1). The group separation is visualized in the cross-validated score plot shown in Figure 2D, and the 11 selected metabolites are shown in Figure 2E. A fair discriminatory ability with an AUC of 0.73 was observed for the PD group, and a very good discriminatory ability with an AUC of 0.91 was observed for the PSP group (Figure 2F). Ten of these eleven metabolites had a univariate *p*-value below 0.05 (Tables S4 and S5).

In the OPLS-DA of the MSA and PSP groups, the model lacked statistical significance (Q^2^ = 0.206, *p* = 0.802). By selecting metabolites with |w*| > 0.175, a valid model was obtained (Q^2^ = 0.626, *p* = 1.2 × 10^−5^; Table 1). The group separation is visualized in the cross-validated score plot in Figure 2G, and selected metabolites are shown in Figure 2H. A good discriminatory ability was observed for the PSP group with an AUC of 0.89, but for the MSA group an AUC of 0.56 indicated very poor discriminatory ability (Figure 2I). Among these nine metabolites, five had a *p*-value below 0.05 according to Student’s *t*-test (Tables S4 and S5).

A schematic summary of the metabolites responsible for the discrimination between groups, as well as the physiological significance for each metabolite, is shown in Table 2.

## 3. Discussion

The present study is, to our knowledge, the first to combine NMR spectroscopy and MS spectrometry to quantitatively characterize and compare the plasma metabolomes of patients with PD and the atypical Parkinsonian disorders MSA and PSP as well as of those of matched control subjects. By combining the two methods, increased coverage of the metabolome is obtained, where NMR and MS focus preferentially on soluble and lipid metabolites, respectively, with only a minor overlap of metabolites. Multivariate statistical analysis showed significant differences in the metabolic profiles of the different groups, as will be discussed below. Because the differential diagnosis between PD, MSA, and PSP is difficult due to similar clinical diagnostic features, especially at early stages of the diseases, these observations may have an impact on future diagnosis and treatment procedures and may provide important clues to the underlying disease mechanisms.

### 3.1. PD Patients versus Control Subjects

In the multivariate analysis of the merged NMR and MS data, the metabolic profile of PD was significantly different from the control group (*p* = 1.8 × 10^−5^), where seven metabolites were particularly important for discrimination (Figure 1, Table 1 and Table 2, Tables S4 and S5). In comparison to controls, a reduced concentration of formic acid (*fc* = 0.83, *p* = 0.0066) and an increased concentration of succinate (*fc* = 1.23, *p* = 0.001) were observed. Formic acid is involved in pyruvate metabolism, methane metabolism and is a product in the kynurenine pathway, which is known to be affected in PD [33,34,35]. However, a previous study reported an increased level of formic acid in the PD group compared to the control group [30]; their use of unfiltered serum and use of a different NMR technique, may contribute to these conflicting results. Succinate acts as a substrate in the citric acid cycle, where oxidation by succinate dehydrogenase is coupled to the electron transfer to ubiquinone in the mitochondrial respiratory chain. Consequently, an increased level of succinate may be linked to mitochondrial dysfunction, neurodegeneration, and PD [36]. The citric acid cycle metabolites pyruvate and citrate were quantified but did not show any statistically significant alterations. Furthermore, based on the NMR data, PD patients have elevated concentrations of arginine (*fc* = 1.34, *p* = 0.0439) and reduced levels of lysine (*fc* = 0.74, *p* = 0.0286). Arginine is part of the arginine and proline metabolism pathway and is a substrate for nitric oxide synthase [37,38], which links arginine to the regulation of oxidative stress. However, a previous study using plasma did not observe any changes in arginine concentration [21], and a study in serum found a reduced concentration [29]; the use of different methods, liquid chromatography compared to NMR spectroscopy, may contribute to the observed discrepancies. An increased level of arginine was previously observed in mildly cognitively impaired and Alzheimer’s disease patients [39], indicating possible similarities between these patients and those with PD. A reduced concentration of lysine in PD patients compared to control subjects was previously observed in both the CSF and serum/plasma [21,29]. Lysine is essential for humans, and the neurotransmitter glutamate is formed through the lysine degradation pathway [40]. However, the MS data showed no clear change in arginine (*fc* = 0.97, *p* = 0.554) and an elevated level of lysine (*fc* = 1.1, *p* = 0.023). The observed differences may be caused by the different sample preparation procedures in the NMR and the MS analysis. Taken together, it suggests a need for a conservative interpretation of the results for arginine and lysine.

The three additional discriminatory metabolites were identified from the MS data, and among them carnitine showed an elevated level (*fc* = 1.28, *p* = 0.00007) in the PD group. Carnitine is synthesized endogenously from lysine in the carnitine biosynthetic pathway, which thereby links lysine and carnitine metabolically. Carnitine is further involved in the shuttling of long-chain fatty acids into the mitochondria for β-oxidation and energy production [41]. Contradictory results have been reported, with either a reduced level of carnitine and disrupted glycerol phospholipid metabolism [23] or an unaltered level of carnitine [28]. In this study, reduced levels of carnitine were observed for lysophosphatidylcholine (lysoPC) a C26:0 (*fc* = 0.52, *p* = 0.00006) and lysoPC a C28:1 (*fc* = 0.59, *p* = 0.00002). The shorter lipid, lysoPC a C26:0, has been shown to be a marker for peroxisome disorders [42], while altered levels of the longer lipid, lysoPC a C28:1, have not previously been observed. Because both are glycerophospholipids, our observations suggest a perturbation in glycerophospholipid metabolism within the PD group. A similar disturbance of this metabolic pathway was previously observed for a PD group, where an increased level of a shorter lipid variant, lysoPC a C20:0, was detected [43]. Furthermore, altered PC/lysoPC ratios were previously observed for PD patients [44]. 

### 3.2. MSA Patients versus Control Subjects

In the comparison between MSA and control subjects, a statistically significant model was obtained (*p* = 0.007, Table 1) and seven metabolites were particularly affected (Figure 1, Table 2, Tables S4 and S5). The concentrations of formic acid (*fc* = 0.83, *p* = 0.0128) and succinate (*fc* = 1.21, *p* = 0.0038) were altered similarly as in the PD group, as discussed above. Similar to the PD group, no statistically significant alterations were observed for the citric acid cycle metabolites pyruvate and citrate. A significantly reduced level of taurine (*fc* = 0.81, *p* = 0.0123) was observed in the NMR data for the MSA group when compared to the control group. Taurine is a highly abundant metabolite in mammals and serves various physiological functions, for example, as a neurotransmitter or as a neuro-protective agent [45]. Although not previously reported for MSA patients, studies have shown reduced levels of taurine in PD patients, both in the CSF and in plasma [46,47]; however, the present study did not identify an altered level of taurine in the PD group. The MSA group further exhibited an elevated concentration of lactate (*fc* = 1.24, *p* = 0.0147), a metabolite that is closely linked to formic acid via pyruvate metabolism and is involved in glucose metabolism [48]. The multivariate analysis also identified trimethylamine N-oxide (TMAO) as a discriminatory metabolite (*fc* = 1.19, *p* = 0.0577), and TMAO is linked metabolically to formic acid via methane metabolism.

Increased concentrations of the two phosphatidylcholine (PC) metabolites, PC ae C42:4 (*fc* = 1.21, *p* = 0.0256) and PC ae C44:5 (*fc* = 1.21, *p* = 0.0442), were detected in the MSA group when compared to the control group. This indicates a disturbance in lipid metabolism, and such a disturbance has not previously been observed, although alterations of PC ae C42:4 have been associated with the aggregation of amyloid-β in Alzheimer’s disease [49].

### 3.3. PSP Patients versus Control Subjects

When the PSP group was compared to the control group, a statistically significant model was obtained (*p* = 4.1 × 10^−6^, Table 1) where nine discriminatory metabolites were identified (Figure 1, Table 2, Tables S4 and S5), among them the previously discussed formic acid (*fc* = 0.53, *p* = 0.0000), succinate (*fc* = 1.21, *p* = 0.0046), arginine (*fc* = 1.51, *p* = 0.0223), carnitine (*fc* = 1.56, *p* = 0.0000), taurine (*fc* = 0.85, *p* = 0.0237), and PC ae C44:5 (*fc* = 1.27, *p* = 0.0036). Formic acid and succinate showed similar changes as in the PD and MSA groups. The changes in arginine and carnitine were similar to the PD group, while the altered levels of taurine and PC ae C44:5 were similar to those of the MSA group. Interestingly, an increased level of arginine was observed in mildly cognitively impaired/Alzheimer’s disease patients [39], suggesting a link between the two tauopathy disorders of Alzheimer’s disease and PSP. Sarcosine, which is involved in glycine, serine, and threonine metabolic pathway as well as in the arginine and proline metabolic pathway, was reduced in the PSP group (*fc* = 0.66, *p* = 0.00001). Because both sarcosine and arginine are part of the latter pathway, their altered levels suggest a disturbance of this pathway. A reduced level of dimethylamine was also observed in the PSP group (*fc* = 0.76, *p* = 0.0013), which was similar to previous studies in CSF that have reported lowered levels of dimethylamine in PD patients [20,21,29,33]. Dimethylamine is involved in methane metabolism, where dimethylamine is formed from trimethylamine by dimethylamine/trimethylamine dehydrogenase [50]. Dimethylamine can also be produced from asymmetric dimethylarginine, an endogenous inhibitor of nitric oxide synthase [51]. Dimethylamine and arginine, which are substrates of nitric oxide synthase [37,38], are thus linked to the regulation of oxidative stress. An increased level of pyruvic acid (*fc* = 1.23, *p* = 0.0109) was observed, and pyruvic acid plays a major role as an energy source in the citric acid cycle in the mitochondria, and the increased concentration suggests altered pyruvic acid metabolism [52]. 

### 3.4. PD versus MSA Patients

In the comparison between PD and MSA patients, a significant discriminatory model was obtained using a selection of metabolites (*p* = 1.0 × 10^−4^, Table 1). Several of these metabolites displayed elevated concentrations in the PD group (Figure 2, Table 2, Tables S4 and S5), among them the previously discussed metabolites arginine (*fc* = 1.45, *p* = 0.0385) and taurine (*fc* = 1.26, *p* = 0.0056). PD patients also had higher levels of acetylcarnitine (*fc* = 1.35, *p* = 0.0189), a metabolite with several important roles in metabolism, in the mitochondrial fatty-acid shuttling system and in β-oxidation and that acts as a precursor for acetylcholine and is linked to glutamate, glutamine, and GABA synthesis [53,54,55]. Interestingly, both glutamate (*fc* = 1.38, *p* = 0.0280) and glutamine (*fc* = 0.95, *p* = 0.1740) were identified as discriminatory metabolites. Glutamine and glutamate are tightly linked metabolically, and they are also involved in arginine biosynthesis. Furthermore, glutamate is linked to the discriminatory metabolite glycine (*fc* = 0.78, *p* = 0.01634) via glutathione metabolism, as well as indirectly to taurine through their interaction with common receptors [56]. Elevated levels of the two metabolites acetoacetate (*fc* = 1.81, *p* = 0.1205) and 3-hydroxybutyrate (*fc* = 1.94, *p* = 0.1100) were observed in the PD group, suggesting a difference in the MSA group regarding ketone body metabolism. The higher level of 2-hydroxybutyrate (*fc* = 1.26, *p* = 0.027) in the PD group compared to the MSA group suggests increased lipid oxidation and oxidative stress, as previously described in a study of insulin resistance [57]. The discriminatory metabolite 3-hydroxyisobutyrate (*fc* = 1.23, *p* = 0.551) was previously shown to affect enzyme activities related to brain metabolism homeostasis and neurotransmission, where an increased level of 3-hydroxyisobutyrate, as seen in the present study, may contribute to neurodegeneration [58]. The remaining discriminatory metabolite, creatinine, was increased (*fc* = 1.25, *p* = 0.0472) in the PD group compared to the MSA group, suggesting a difference in creatine/creatinine metabolism.

### 3.5. PD versus PSP Patients

When the PD group was compared to the PSP group, a significant model was obtained (*p* = 9.9 × 10^−7^, Table 1) using a selected set of metabolites. Altered levels were observed for six of the previously discussed discriminatory metabolites, including formic acid (*fc* = 1.57, *p* = 0.0016), taurine (*fc* = 1.19, *p* = 0.0106), sarcosine (*fc* = 1.38, *p* = 0.0004), dimethylamine (*fc* = 1.26, *p* = 0.0245), lysine (*fc* = 0.75, *p* = 0.0451), and carnitine (*fc* = 0.82, *p* = 0.0079). The PD group further had higher levels of histidine (on both instrument platforms: NMR: *fc* = 1.12, *p* = 0.0320, MS: *fc* = 1.15, *p* = 0.0062) and valine (*fc* = 1.16, *p* = 0.0120), while reduced levels were detected for glucose (*fc* = 0.92, *p* = 0.2157) and lysoPC a C28:0 (*fc* = 0.76, *p* = 0.0050). Changes in the levels of histidine, valine, and glucose were previously identified in the CSF of PD patients [19]. Alterations in the histidine level may suggest differences in neurotransmission because histidine is a precursor of histamine. The changes in valine indicate a possible difference in branched-chain amino acid metabolism and neurotransmitter synthesis, while changes in glucose suggest differences in glucose metabolism. Finally, the decreased level of lysoPC a C28:0 indicated that the effects on lipid metabolism in the PD group were different from the effects in the PSP group.

### 3.6. MSA versus PSP Patients

The metabolic profile of the MSA group was clearly different from that of the PSP group (*p* = 1.2×10^−5^, Table 1) using a selection of metabolites. Seven metabolites were particularly important for discrimination between the MSA and PSP groups (Figure 2, Table 2, Tables S4 and S5), and all of these discriminatory metabolites were identified and discussed in the previous analysis. The MSA group showed increased levels of formic acid (*fc* = 1.56, *p* = 0.0085), sarcosine (*fc* = 1.19, *p* = 0.0188), dimethylamine (*fc* = 1.18, *p* = 0.0911), glycine (*fc* = 1.21, *p* = 0.1165), and histidine (*fc* = 1.07, *p* = 0.0600) and reduced levels of arginine (*fc* = 0.61, *p* = 0.0081), carnitine (*fc* = 0.68, *p* = 0.0006), acetylcarnitine (*fc* = 0.64, *p* = 0.0037), and acetoacetate (*fc* = 0.61, *p* = 0.0748) when compared to the PSP group.

### 3.7. Discriminatory Metabolites and Disease Mechanisms

Although the three diseases of PD, MSA, and PSP all display similar clinical symptoms at an early disease stage, the effects on the metabolome are dissimilar (Table 2), and each disease group has a distinct set of metabolites that can separate the disease group from the control group. Only two metabolites, formic acid and succinate, showed comparable changes in all three disease groups. Surprisingly these metabolites are the only two shared by the α-synucleinopathies PD and MSA. As discussed above, these metabolites are linked to pathways known to be affected in Parkinsonian disorders; formic acid is involved in pyruvate metabolism, methane metabolism and linked to the kynurenine pathway, while succinate is part of the citric acid cycle and is linked to mitochondrial dysfunction. 

In the comparison with the control group, both PD and PSP shared the discriminatory metabolites carnitine and arginine, and this suggests a common disturbance in the carnitine biosynthesis pathway or alternatively in the mitochondrial shuttling system or in arginine metabolism. When comparing the altered metabolites in the MSA and PSP groups, similar changes were observed for the neurotransmitter/protector taurine and the lipid PC ae C44:5.

### 3.8. Concluding Remarks

In the present study, distinct differences in metabolite concentrations were detected in blood plasma from patients suffering from different types of Parkinsonian disorders (PD, MSA, and PSP) and from control subjects (Table 2). This provides an important basis for developing a blood-based panel of metabolite biomarkers that can be used in the clinic to diagnose PD, MSA, and PSP and to differentiate between these clinically similar disorders. These findings are based on comparisons between groups with only a moderate number of well-matched patients and controls, and thus need to be further validated in larger and ideally longitudinal patient cohorts. The study further identifies a limited number of affected metabolic pathways for each disease, providing details about the underlying disease mechanisms that can lead to suggestions about druggable target proteins.

## 4. Materials and Methods

### 4.1. Participants and Sample Collection

Participants were selected from the neurobiobank at the Department of Clinical Sciences, Neurosciences, Umeå University Hospital, Umeå, Sweden. This biobank provided plasma samples from patients diagnosed by two movement disorder specialists according to established consensus criteria for definite PD [59], possible or probable MSA [60] (Parkinsonian phenotype), and possible or probable PSP [61] (Parkinsonian phenotype). The samples were collected at the time of diagnosis before starting dopaminergic treatment. However, during the data analysis process it was discovered that one PD patient and two PSP patients had received levodopa treatment prior to sample collection, and these patients were removed from the statistical analysis. The selected age and gender-matched control subjects included the patient’s spouses and volunteers free of neurological and psychiatric illnesses. All patients had pathological single-photon emission computed tomography scans at the time of diagnosis, while the control individuals displayed normal scans. Table 3 shows a summary of the group characteristics. The limited number of MSA and PSP patients reflects the low incidence of these disorders, when compared to PD. Plasma was collected from venous blood according to a standard operating procedure in which ethylenediamine-tetraacetic acid was used as the anti-coagulant [62]. Samples were stored at –80° C until analysis, at which time they were thawed at room temperature and then gently shaken to avoid any gradient effects.

### 4.2. NMR Analysis

For the NMR analysis, 100 µL of thawed plasma was mixed with 100 µL of 100 mM sodium phosphate buffer pH 7.4 (Scharlau, Germany), transferred into a pre-cleaned Amicon Ultra-0.5 filter device with a 3 kDa cutoff (Merck Millipore, Darmstadt, Germany), and immediately centrifuged at 14,000× *g* for 60 min at 4 °C. A volume of 160 µL of the filtrate solution was mixed with 40 µL NMR buffer solution (5×), resulting in a 200 μL sample containing 50 mM sodium phosphate buffer, pH 7.4, 10% D_2_O, 0.5 mM sodium-3-trimethylsilylpropionate-2,2,3,3-D_4_ (TMSP; Cambridge Isotope Laboratories, Andover, MA, USA), and 0.004% NaN_3_. Each sample was subsequently transferred into a 3 mm NMR tube (Bruker Biospin). The filter cleaning (removing the glycerol) was performed by filtering 0.5 mL Milli-Q H_2_O five times and 0.5 mL 50 mM sodium phosphate buffer, pH 7.4, three times at 14,000× *g* and 36 °C.

All NMR experiments were performed at 298 K on a Bruker Avance III 600 MHz spectrometer equipped with an HCP z-gradient cryo-probe and a cooled SampleJet autosampler (Bruker Biospin). One-dimensional (1D) ^1^H-NMR spectra were acquired using a 1D NOESY (Nuclear Overhauser Effect SpectroscopY) pulse sequence with a relaxation delay of 1.1 s and a mixing time of 100 ms. Water suppression was achieved using excitation sculpting with gradients. Each spectrum consisted of 128 free induction decays (FIDs) collected into 64 K complex data points with a spectral width of 8403.361 Hz and an acquisition time of 3.89 s.

Prior to Fourier transformation, the FIDs were zero-filled to 128 K points and multiplied by an exponential line-broadening function of 0.3 Hz. The 1D spectra were manually phased and baseline corrected, and the chemical shifts were internally referenced to the TMSP signal at 0.0 ppm. TopSpin version 2.1 or 3.0 was used for spectrometer control and data processing (Bruker Biospin, Fällanden, Switzerland). 

Processed spectra were imported into the Chenomx NMR Suite software, version 8 (Chenomx Inc., AB, Edmonton, Canada) in which metabolites were quantified using the targeted profiling approach where individual NMR resonances of interest were mathematically modeled from pure standard metabolite compound spectra stored in an internal database. The internal TMSP resonance was used as the reference.

### 4.3. MS Analysis

The MS-based metabolomics analysis was performed using the Absolute*IDQ^®^* p180 kit from BIOCRATES Life Sciences AG (Innsbruck, Austria), which allows for the quantitative targeted analysis of up to 188 metabolites. The sample preparation was carried out at room temperature according to the manufacturer’s protocol. In brief, 10 μL of thawed plasma, together with an internal standard mix, was applied to a 96-well filter plate followed by a drying period in a Porvair MiniVap nitrogen evaporator (Porvair Sciences Ltd., Wrexham, UK), derivatization, a second drying period, extraction, and filtration. The obtained flow-through was subsequently divided and diluted into two separate 96-well plates for analysis using liquid chromatography (LC) MS and flow injection analysis MS, respectively.

The MS analysis was performed in accordance with the Absolute*IDQ^®^* p180 kit protocol using a Sciex Triple Quad 6500+ LC-MS/MS system (AB Sciex LLC, Framingham, MA, USA) equipped with a Shimadzu ultra-high performance liquid chromatography (UHPLC) system consisting of two Nexera X2 LC-30AD binary pumps and a Nexera X2 SIL 30-AC autosampler combined with a CTO-20AC column oven (Shimadzu Corporation, Kyoto, Japan). In the UHPLC analysis, the samples were analyzed at 50 °C using an ACQUITY UPLC BEH C18 1.7 µm column, 2.1 mm × 75 mm, and an ACQUITY UPLC BEH C18 1.7 µm VanGuard pre-column, 2.1 mm × 5 mm (Waters, Milford, USA). The columns, gradients, and mobile phases used for the analyses are specified in the Absolute*IDQ^®^* p180 kit protocol. Spectral peaks were integrated, and analyte concentrations were determined in the Analyst software (AB Sciex LLC, Framingham, MA, USA), and the resulting data were imported into MetIDQ (version Carbon, BIOCRATES Life Sciences AG, Innsbruck, Austria) for quality assessment and for determining the final list of metabolite concentrations.

### 4.4. Statistical Analysis

The multivariate data analyses and the receiver operating characteristic curve (ROC) analysis of the quantified NMR and MS data sets were carried out using the SIMCA software package (version 16, Umetrics; Umeå, Sweden). The data sets were imported into SIMCA, mean-centered, scaled using unit variance, and subsequently analyzed using unsupervised PCA and supervised OPLS-DA [63,64]. The NMR and MS data sets were analyzed separately as well as together, and the combined analysis was performed by using soft block scaling for the two data sets. The quality of the models was determined by the goodness of fit in the X (R^2^X) and Y (R^2^Y) variables and the predictive ability Q^2^Y. Derived OPLS-DA models were cross-validated by using the seven-fold cross-validation method, and group separations were evaluated through CV-ANOVA [65] and the first weight vector (w*[1]). Cut-off values of w*[1] were determined empirically by iterative model building to obtain the most statistically significant model (according to CV-ANOVA). Univariate analysis was performed using Student’s *t*-tests, and pathway analysis was carried out using the Metaboanalyst web server [66].

## Figures and Tables

**Table 1:** Summary of the statistics for the pairwise OPLS-DA models used to describe group differences between the different disease and control groups. The analysis was based on the merged NMR and MS data.

Comparison	Variables	A_pred_	A_orth_	R^2^X	R^2^Y	Q^2^Y	CV-ANOVA	AUC
PD:Control	All	1	2	0.290	0.801	0.417	1.8 × 10^−5^	0.76:0.98
MSA:Control	All	1	1	0.246	0.753	0.302	0.007	0.89:0.90
PSP:Control	All	1	1	0.248	0.811	0.524	4.1 × 10^–6^	0.91:0.89
PD:MSA	Selected	1	0	0.243	0.430	0.355	1.0 × 10^–4^	0.70:0.87
PD:PSP	Selected	1	0	0.236	0.546	0.467	9.9 × 10^–7^	0.73:0.91
MSA:PSP	Selected	1	1	0.301	0.717	0.626	1.2 × 10^–5^	0.56:0.89

A_pred_, number of predictive components; A_orth_, number of orthogonal components; R^2^X and R^2^Y, the explained variation in X and Y, respectively; Q^2^Y*,* the quality and predictive power of the model. CV-ANOVA is a significance testing based on ANOVA of the cross-validated residuals. AUC is the area under the curve in a ROC analysis and represents the ability to discriminate between groups.

**Table 2:** Summary of the NMR and MS results. Metabolites in plasma important for separation of the PD, MSA and PSP patient groups from the control group, and from each other, were identified in the multivariate statistical models generated from the NMR and the MS data. Increased and decreased concentrations of the metabolites are indicated with an upward or downward arrow, respectively. Bold arrows indicate metabolites with a *p*-value below 0.05 in the univariate analysis. A selection of affected pathways and/or associated physiological functions for each metabolite is listed.

Metabolite	PD	MSA	PSP	PD	PD	MSA	Pathway or Physiological Function
		vs. Control		MSA	vs. PSP	PSP	
Formic acid	🡇	🡇	🡇	🡅	🡅	🡅	Pyruvate met. Methane met., Kynurenine pathway
Succinate	🡅	🡅	🡅				Citric acid cycle
Carnitine	🡅		🡅		🡇	🡇	Carnitine biosynthesis, Mitochondrial shuttle system
L-Arginine^NMR^	🡅		🡅			🡇	Arg and Pro metabolism, linked to oxidative stress
L-Lysine^NMR^	🡇				🡇		Carnitine biosynthesis, Lys synthesis/degradation
lysoPC a C26:0	🡇						Lipid metabolism
lysoPC a C28:1	🡇						Lipid metabolism
Taurine^NMR^		🡇	🡇	🡅	🡅		Neurotransmitter, Neuroprotective
Lactic acid		🡅					Anaerobic glucose and pyruvate metabolism
PC ae C44:5		🡅	🡅				Lipid metabolism
PC ae C42:4		🡅					Lipid metabolism
TMAO		↑					Methane metabolism
Sarcosine			🡇		🡅	🡅	Arg/Pro metabolism, Gly/Ser/Thr metabolism
Dimethylamine			🡇		🡅	↑	Methane metabolism. Link to oxidative stress regulation
Pyruvic acid			🡅				Energy source for citric acid cycle
Acetylcarnitine				🡅		🡇	MCH FA shuttle system, Glu/Gln/GABA synthesis
Glycine^MS^				🡇		↑	GSH metabolism
Acetoacetate				↑		↓	Ketone body, propanoate and Leu metabolism
2-HB				🡅			Lipid oxidation, oxidative stress
3-HB				↑			Ketone body metabolism
3-HIB				↑			Linked to brain metabolism and neurotransmission
Creatinine^MS^				🡅			Creatine phosphate metabolism
Glutamate^MS^				🡅			Glu/Gln/GABA synthesis, Arg synthesis, GSH met.
L-Glutamine^NMR^				↓			Glu/Gln/GABA synthesis, Arg synthesis
L-Histidine^NMR^					🡅	↑	Link to neurotransmission and oxidative stress
L-Histidine^MS^					🡅		Link to neurotransmission and oxidative stress
L-Valine^MS^					🡅		BCAA synthesis, neurotransmitter synthesis
lysoPC a C28:0					🡇		Lipid metabolism
Glucose					↓		Glucose metabolism

Abbreviations: met.: metabolism; lysoPC: lysophosphatidylcholine; PC: phosphatidylcholine ; ^NMR^: based on NMR-data; ^MS^: based on MS-data; TMAO: trimethylamine N-oxide; HB: hydroxybuturate; HIB: hydroxyisobutyrate; MCH: mitochondria; FA: fatty acid; GABA: gamma-aminobutyric acid; GSH: glutathione; BCAA: branched chain amino acids.

**Table 3:** Demographic and clinical characteristics of patients and control subjects.

	Control	PD	MSA	PSP
Number of subjects	31	34	12	17
Gender, Male/Female	17/14	19/15	8/4	9/8
Age, mean ± SD	68.2 ± 6.6	69 ± 6.8	74.4 ± 9.2	75.2 ± 7.3
Levodopa treatment	0	1	0	2

PD = Parkinson’s disease; MSA = multiple system atrophy; PSP = progressive supranuclear palsy.

## Data Availability

Data is contained within the article or Supplementary Materials.

## Supplementary Materials

The following are available online at https://www.mdpi.com/article/10.3390/metabo11120860/s1, Supplementary results; Table S1: Quantified metabolites using NMR, Table S2: Quantified metabolites using MS, Table S3: Model statistics, Table S4: Uni- and multivariate statistical analysis of NMR-detected metabolites in plasma, Table S5: Uni- and multivariate statistical analysis of MS-detected metabolites in plasma, Table S6: Summary of the NMR results, Table S7: Summary of the MS results, Figure S1: Multivariate statistical comparisons between the patient groups and control group, using the NMR data, Figure S2: Multivariate statistical comparisons between the patient groups, using the NMR data, Figure S3: Multivariate statistical comparisons between the patient groups and control group, using the MS data, Figure S4: Multivariate statistical comparisons between the patient groups, using the MS data.
