# Supplementary material for: Plasma Metabolite Markers of Parkinson’s Disease and Atypical Parkinsonism
Source: Metabolites. 2021 Dec 9;11(12):860. doi: 10.3390/metabo11120860 (PMC8706715; doi:10.3390/metabo11120860)
Supplement: Supplementary file 1 [file metabolites-11-00860-s001.zip › metabolites-1488248-supplementary.pdf]

Supplemental material

Plasma metabolite markers of Parkinson's disease and atypical Parkinsonism

Meerakhan Pathan[1], Junfang Wu[1,2], Hans-Åke Lakso[3], Lars Forsgren[4] and Anders Öhman[1,]*

[1]Department of Integrative Medical Biology, Umeå University, SE - 901 87 Umeå, Sweden,
[2]Division of Cardiology, Department of Internal Medicine, Tongji Hospital, Tongji Medical College, Huazhong University of Science and Technology, Wuhan 430030, China,
[3]Department of Medical Biosciences, and [4]Department of Clinical Sciences, Neurosciences, Umeå University, SE - 901 85 Umeå, Sweden

*__Corresponding author__:

Anders Öhman
Department of Integrative Medical Biology
Umeå University
SE – 901 87 Umeå, Sweden
E-mail: anders.ohman01@umu.se
Phone: +46 (0)90 786 7211

Keywords: Parkinson's disease, multiple system atrophy, progressive supranuclear palsy, nuclear magnetic resonance, mass spectrometry, metabolomics, atypical Parkinsonism, plasma, biomarker

Results

NMR-based analysis

Table S1 shows the average metabolite concentrations in the different groups as determined by NMR. An overview of the statistical analysis of the NMR data is presented in the upper part of Table S2 and will be described in detail below.

For the PD and control groups, the OPLS-DA was able to separate the two groups but did not provide a statistically significant model ($Q^2 = 0.05$, $p = 0.542$). However, by selecting the nine most important metabolites (those with weight values $|w^*| > 0.2$) a good model was obtained ($Q^2 = 0.33$, $p = 7.9 \cdot 10^{-5}$; Table S3), see the resulting cross-validated score plot in Fig. S1A and the selected metabolites in Fig. S1B. The accompanying ROC analysis is shown in Fig. S1C and illustrates a modest discriminatory ability with an AUC of 0.69. A univariate analysis using Student's t-test showed that four of the identified metabolites had a *p*-value below 0.05. The fold changes, *p*-values (from the univariate analysis), and weight values (from the multivariate analysis) for all metabolites are listed in Table S4.

In the OPLS-DA of the MSA and control groups, a statistically poor separation ($Q^2 = 0.078$, $p = 0.198$) was observed. The nine most important metabolites of this initial model (with $|w^*|>0.2$) were used in a second round of modeling that resulted in a statistically significant model ($Q^2 = 0.298$, $p = 0.0080$; Table S3), see the cross-validated score plot in Fig. S1D and selected metabolites in Fig. S1E. The ROC analysis, shown in Fig. S1F, indicates a fair discriminatory ability with an AUC of 0.74. Four of these nine metabolites had a *p*-value below 0.05 in the univariate analysis (Table S4).

When PSP patients were compared with controls, a statistically significant OPLS-DA model ($Q^2 = 0.374$, $p = 0.0006$) was obtained. One of the patients did, however, clearly overlap with the control group. This was the only PSP-pagf patient in the cohort, and removing this single patient from the analysis resulted in an even stronger model ($Q^2 = 0.472$,

$p = 3.0 \cdot 10^{-5}$; Table S3). The cross-validated score plot of this model is shown in Fig. S1G, and metabolites with |w*| above 0.2 are shown in Fig. S1H. According to the ROC analysis, the model had a good discriminating ability with an AUC of 0.86, see Fig S1I. All of the identified metabolites were also of importance in the univariate analysis ($p < 0.05$).

Furthermore, pair-wise OPLS-DA was utilized to discriminate the different disease groups from each other. In the comparison between PD and MSA, a significant model was not obtained ($Q^2 = 0.109$, $p = 0.109$). By selecting the most important metabolites (with |w*| > 0.2) from that model, a second round of modeling gave a statistically significant model ($Q^2 = 0.221$, $p = 0.0052$; Table S3). Figures S2A and S2B show the cross-validated score plot of this model and the |w*| of the nine retained metabolites, respectively. ROC analysis showed a fair discriminatory ability with an AUC of 0.71 for the PD group and 0.79 for the MSA group, see Fig. S2C. Four out of these nine metabolites had a *p*-value below 0.05 according to Student's t-test.

In the initial OPLS-DA of the PD and PSP groups, it was apparent that the single PSP-pagf patient severely disturbed the modeling, and this patient was therefore removed from the analysis. A statistically significant separation was thereafter obtained ($Q^2 = 0.140$, $p = 0.036$; Table S3), see the cross-validated score plot in Fig. S2D. Ten metabolites had a |w*| > 0.2 and therefore were considered the most important, see Fig. S2E, and seven of these also had a *p*-value below 0.05 in the univariate analysis. According to the ROC analysis, the discriminatory ability was fair to good for the PD and the PSP groups, with an AUC of 0.70 and 0.84, respectively, (Fig. S2F).

In the OPLS-DA of the MSA and PSP groups, the single PSP-pagf patient caused similar problems as described above and was therefore removed. The subsequent model did, however, lack statistical significance ($Q^2 = 0.187$, $p = 0.847$). By selecting metabolites with weight values (|w*|) above 0.2, a valid model was obtained ($Q^2 = 0.339$, $p = 0.0085$; Table

S3). The group separation is visualized in the cross-validated score plot in Fig. S2G, and selected metabolites are shown in Fig. S2H. A modest discriminatory ability with an AUC of 0.64 was observed for the MSA group, and a fair discriminatory ability with an AUC of 0.77 was observed for the PSP group, see Fig. S2I. Among these six metabolites, only two had a *p*-value below 0.05 according to Student's t-test.

The parameters used in the OPLS-DA modeling, the CV-ANOVAs, and the AUCs of each pair-wise comparison are listed in Table S3. Each metabolite's fold change, weight value, and *p*-value are displayed in Table S4. A schematic summary of the metabolites responsible for the discrimination between groups is shown in Table S6.

MS-based analysis

Table S2 shows the average metabolite concentrations in the different groups as determined by MS. The parameters used in the OPLS-DA modeling, the CV-ANOVAs, and the AUCs of each pair-wise comparison are listed in Table S3. The results obtained from the statistical analysis of the MS data are described in detail below.

In the OPLS-DA comparing PD and control samples, an excellent model was obtained ($Q^2 = 0.396$, $p = 4.5 \cdot 10^{-7}$; Table S3). The cross-validated score plot is displayed in Fig. S3A, and the 14 most important metabolites (|w*| larger than 0.15) are shown in Fig. S3B. All of these metabolites had a Student's t-test significance below 0.05 (Table S5). The ROC analysis gave an AUC of 0.78, which corresponds to a fair discriminatory ability, see Fig. S3C.

The OPLS-DA between the MSA and the control groups also resulted in a statistically significant model ($Q^2 = 0.352$, $p = 0.002$; Table S3), see the cross-validated score plot in Fig. S3D. Fig. S3E shows 10 metabolites with |w*| larger than 0.15, where all had a *p*-value below 0.05 in the univariate analysis (Table S5). A very good discrimination between groups was detected by the ROC analysis, with an AUC of 0.96, see Fig. S3F.

The PSP and control groups were also clearly separated according to the OPLS-DA (Q^2 = 0.409, p = 0.00025; Table S3), see the score plot in Fig. S3G and the seven most significant metabolites (|w*| > 0.15) in Fig. S3H. These metabolites all had a Student's t-test p-value below 0.05 (Table S5). The results of the ROC analysis (AUC = 0.89) are shown in Fig. S3I and suggests a good discriminative ability.

In the OPLS-DA of the PD and MSA groups, the obtained model lacked statistical significance (Q^2 = 0.260, p = 0.164). Metabolites with |w*| above 0.15 were selected, and a valid model was obtained (Q^2 = 0.277, p = 0.0011; Table 3S). The group separation is visualized in the cross-validated score plot shown in Fig. S4A, and the 11 selected metabolites are shown in Fig. S4B. A modest discriminatory ability with an AUC of 0.64 was observed for the PD group, and a good ability with an AUC of 0.89 was observed for the MSA group, see Fig. S4C. Nine of these metabolites had a p-value below 0.05 (Table S5).

In the OPLS-DA between the PD and PSP groups, a statistically significant model was obtained (Q^2 = 0.388, p = 0.034; Table S3) that provided good group separation, see Fig. S4D. The 10 most important metabolites (|w*|>0.15) are shown in Fig. S4E, and the ROC analysis is shown in Fig. S4F. Seven of these metabolites are of importance in the univariate analysis (p < 0.05; Table S5). The discriminative ability was fair for PD (AUC = 0.79) and good for PSP (AUC = 0.84).

In the comparison of MSA and PSP, the OPLS-DA did not result in a statistically significant model (Q^2 = 0.151, p = 0.465). However, by selecting metabolites with a |w*| above 0.15 within the initial model, 10 metabolites were utilized in a second round of modeling, resulting in a valid model (Q^2 = 0.571, p = 0.00097; Table S3). The score plot, the metabolites, and the ROC analysis are shown in Figures S4G–I. The discrimination between groups was fair for both MSA (AUC = 0.75) and PSP (AUC = 0.74). Of these 10 metabolites, seven had a p-value below 0.05 in the univariate analysis (Table S5).

The parameters used in the OPLS-DA modeling, the CV-ANOVAs, and the AUCs of each pair-wise comparison are listed in Table S3. Each metabolite's fold change, weight value, and *p*-value are shown in Table S5. A schematic summary of the metabolites responsible for the discrimination between groups is shown in Table S7.

Table S1. **Quantified metabolites using NMR.** A list of the quantified metabolites, their concentration averages, and standard deviations for the respective disease and control groups.

**Metabolite**	**PD**		**MSA**		**PSP**		**Ctrl**	
	Mean *[μM]*	*SD*	*Mean* *[μM]*	*SD*	*Mean* *[μM]*	*SD*	*Mean* *[μM]*	*SD*
2-Hydroxybutyrate	37.1	10.6	29.4	7.7	35.2	15.7	37.3	16.4
2-Hydroxyisobutyrate	0.8	1.2	1.1	1.8	1.7	2.9	0.7	0.8
2-Hydroxyisovalerate	4.5	4.9	4.9	4.3	3.8	3.7	5.7	4.7
3-Hydroxybutyrate	85.6	86.0	44.1	25.9	73.4	86.7	83.8	83.9
3-Hydroxyisobutyrate	16.9	5.3	13.7	3.0	15.5	5.0	14.9	4.0
Acetate	48.9	18.5	42.9	10.5	41.4	9.9	44.5	17.2
Acetoacetate	39.5	37.9	21.9	10.7	35.7	23.7	38.0	30.4
Acetone	15.0	5.5	13.6	2.6	16.8	13.4	14.6	4.1
Alanine	365.7	79.1	355.7	44.6	344.4	89.0	344.4	55.5
Arginine	48.3	23.2	33.4	11.3	54.5	23.1	36.0	24.6
Citrate	159.7	42.7	161.9	36.5	160.8	47.4	158.7	28.8
Creatine	30.2	51.7	22.6	16.8	30.6	19.3	28.9	17.7
Creatinine	87.8	26.4	82.3	18.0	91.1	50.3	77.5	13.1
Dimethylamine	3.7	1.1	3.5	0.6	2.9	0.8	3.9	0.8
Formic acid	53.7	18.3	53.3	16.0	34.2	17.6	64.4	11.1
Glucose	4492.8	788.1	4380.5	650.6	4880.1	1307.3	4402.9	452.4
Glutamate	69.3	21.2	60.7	22.1	68.0	30.2	59.2	22.9
Glutamine	468.9	49.7	492.1	50.0	455.1	63.9	459.9	48.1
Histidine	76.6	13.1	73.3	4.9	68.3	7.6	71.5	9.0
Isoleucine	63.7	16.8	54.4	6.4	52.4	10.8	58.8	13.4
Lactate	1368.3	508.6	1514.0	424.0	1383.2	643.8	1218.5	305.2
Leucine	112.7	25.8	99.5	13.1	95.5	15.8	106.5	22.6
Lysine	93.2	61.6	111.1	55.0	129.7	36.2	125.8	54.2
Mannose	26.3	14.2	25.9	11.3	31.3	13.9	25.4	12.5
Methionine	20.6	7.0	19.0	4.6	17.0	4.7	18.8	7.9
Phenylalanine	50.5	9.7	47.4	5.4	48.8	7.6	48.2	6.4
Proline	207.6	44.9	191.7	41.6	195.4	50.6	185.8	44.2
Pyruvate	88.1	27.5	86.9	23.5	99.3	25.4	81.0	19.4
Succinate	11.8	3.3	11.6	2.7	11.6	3.0	9.6	1.5
Taurine	139.2	24.3	110.9	39.2	117.4	28.8	137.7	26.1
Threonine	103.3	17.7	102.8	24.4	94.1	20.9	100.3	13.5
Trimethylamine N-oxide	31.4	9.6	36.3	13.3	33.6	10.0	30.5	6.2
Tyrosine	73.8	17.1	67.5	17.7	64.0	14.3	72.8	17.8
Valine	235.8	43.7	211.4	27.6	211.2	41.7	226.8	40.6

Table S2. Quantified metabolites using MS. A list of the quantified metabolites and their concentration averages and standard deviations for the respective disease and control groups.

Metabolite	PD Mean [μM]	PD SD	MSA Mean [μM]	MSA SD	PSP Mean [μM]	PSP SD	Control Mean [μM]	Control SD
Alanine	391.3	79.1	384.9	86.5	409.4	102.7	373.2	76.6
Arginine	79.1	19.2	71.2	14.1	81.4	19.3	81.8	16.7
Asparagine	45.6	9.6	42.0	7.9	43.2	8.5	46.1	9.8
Citrulline	38.8	10.9	34.2	9.8	39.8	10.2	38.4	9.2
Glutamine	731.6	75.1	778.0	119.4	736.3	95.2	710.9	91.5
Glutamate	55.3	21.1	40.1	15.5	54.6	23.4	46.4	20.6
Glycine	250.4	69.1	320.6	115.0	265.1	51.7	288.3	102.0
Histidine	95.5	14.0	92.8	12.8	83.3	11.4	90.7	8.3
Isoleucine	89.1	22.9	72.6	11.9	76.0	13.4	81.0	16.9
Leucine	166.3	38.8	139.7	21.2	145.6	26.2	159.6	32.6
Lysine	266.7	41.0	276.5	40.4	266.0	39.3	242.6	42.0
Methionine	25.3	7.7	24.7	6.2	22.8	4.3	26.4	5.3
Ornitine	95.2	22.8	93.1	13.8	95.3	14.3	82.4	17.5
Phenylalanine	69.8	12.0	64.6	8.2	67.4	12.3	67.6	10.5
Proline	245.9	58.5	229.6	54.5	249.1	81.1	233.2	55.9
Serine	116.7	26.0	113.0	32.8	116.4	26.9	114.1	23.6
Threonine	117.7	25.3	119.1	32.8	112.5	27.3	116.4	16.3
Tryptophan	65.1	13.1	57.4	11.7	57.2	12.2	63.8	8.0
Tyrosine	81.6	18.8	71.6	16.9	69.6	21.2	80.2	18.7
Valine	285.2	49.4	253.9	27.2	245.5	42.7	271.3	43.8
ADMA	0.5	0.1	0.4	0.1	0.5	0.1	0.5	0.1
Creatinine	93.8	29.4	74.8	22.1	89.5	47.6	83.3	16.5
Kynurenine	3.1	0.9	2.5	0.7	3.3	1.6	2.7	0.7
Sarcosine	5.2	1.3	4.5	0.8	3.8	0.7	5.8	1.4
SDMA	0.6	0.1	0.6	0.2	0.6	0.3	0.5	0.1
Serotonin	0.3	0.3	0.2	0.2	0.4	0.4	0.2	0.1
Taurine	84.1	25.6	81.4	30.5	87.8	26.9	–	–
Carnitine	47.9	11.3	39.8	9.2	58.8	14.2	37.6	7.7
Acetylcarnitine	7.4	2.5	5.5	1.4	8.6	3.1	6.3	2.4
Propionylcarnitine	0.5	0.1	0.5	0.1	0.5	0.1	0.4	0.1
Malonylcarnitine	0.1	0.1	0.0	0.0	0.1	0.0	0.0	0.0
Tetradecenoylcarnitine	0.1	0.1	0.1	0.0	0.1	0.1	0.1	0.0
lysoPC a C16:0	78.8	14.6	72.0	15.9	82.0	16.7	82.8	13.7
lysoPC a C16:1	2.4	0.6	2.5	0.9	2.7	0.7	2.4	0.8
lysoPC a C17:0	1.7	0.4	1.5	0.4	1.5	0.5	1.7	0.4
lysoPC a C18:0	23.3	5.1	20.4	3.9	23.4	5.9	25.0	3.9
lysoPC a C18:1	16.7	3.8	17.1	4.3	18.2	3.4	18.1	4.9
lysoPC a C18:2	23.8	7.3	21.8	7.8	23.5	6.9	25.4	9.5
lysoPC a C20:3	1.7	0.5	1.7	0.6	1.9	0.6	1.8	0.5
lysoPC a C20:4	4.0	1.5	3.7	1.1	4.3	0.9	4.1	1.3
lysoPC a C24:0	0.2	0.1	0.1	0.1	0.2	0.1	0.2	0.1
lysoPC a C26:0	0.1	0.1	0.1	0.0	0.2	0.1	0.2	0.1
lysoPC a C26:1	0.1	0.0	0.1	0.0	0.1	0.0	0.2	0.1
lysoPC a C28:0	0.2	0.0	0.2	0.0	0.2	0.1	0.2	0.1
lysoPC a C28:1	0.3	0.1	0.3	0.1	0.3	0.1	0.5	0.2
PC aa C24:0	0.1	0.0	0.1	0.0	0.1	0.0	0.1	0.0
PC aa C28:1	3.9	0.9	3.5	0.9	4.1	1.0	4.2	1.0
PC aa C30:0	5.4	1.9	4.7	1.2	5.3	2.0	5.3	1.7
PC aa C30:2	0.1	0.1	0.1	0.0	0.1	0.1	0.1	0.1
PC aa C32:0	18.3	3.9	17.3	4.1	19.8	5.0	18.6	3.9
PC aa C32:1	21.7	8.9	24.2	12.4	25.2	12.9	23.2	10.0
PC aa C32:2	4.0	1.5	3.7	1.2	4.2	2.0	4.5	1.7
PC aa C32:3	0.6	0.2	0.6	0.2	0.6	0.2	0.6	0.2
PC aa C34:1	291.1	68.1	317.0	99.8	328.8	95.4	299.6	64.0
PC aa C34:2	421.9	80.6	420.9	93.3	458.3	111.8	449.5	74.2
PC aa C34:3	17.9	5.4	18.0	6.2	20.4	6.8	19.9	5.5
PC aa C34:4	1.7	0.7	1.6	0.6	1.7	0.9	1.8	0.6
PC aa C36:0	2.7	0.7	2.2	0.9	2.3	0.7	3.6	1.4
PC aa C36:1	58.9	14.3	63.7	25.6	62.9	22.8	60.8	11.9
PC aa C36:2	251.5	56.4	244.1	41.1	259.6	62.1	273.0	47.8
PC aa C36:3	134.7	27.6	143.3	32.2	155.9	38.7	144.6	27.4
PC aa C36:4	180.6	53.9	172.5	40.8	200.1	58.9	187.0	41.5
PC aa C36:5	44.0	19.2	36.8	17.5	41.5	20.8	56.4	32.1
PC aa C36:6	1.3	0.5	1.1	0.5	1.2	0.6	1.5	0.6
PC aa C38:0	3.5	0.9	3.2	1.2	3.1	0.9	3.7	1.0
PC aa C38:1	0.8	0.6	0.8	0.3	0.9	0.6	1.4	0.7

PC aa C38:3	44.5	10.6	45.3	11.5	50.5	16.7	45.8	9.7
PC aa C38:4	84.8	25.1	81.0	19.8	92.2	29.9	87.2	20.1
PC aa C38:5	60.4	17.0	56.2	14.6	61.0	19.1	65.7	17.6
PC aa C38:6	120.0	34.1	101.3	32.7	111.0	44.2	122.0	28.4
PC aa C40:2	0.3	0.1	0.3	0.1	0.3	0.1	0.4	0.2
PC aa C40:3	0.6	0.2	0.5	0.1	0.6	0.1	0.7	0.2
PC aa C40:4	2.7	0.6	2.8	0.9	3.0	1.4	2.6	0.5
PC aa C40:5	8.9	2.6	8.6	2.7	8.9	3.4	8.9	2.2
PC aa C40:6	34.0	9.8	28.9	9.3	30.9	12.3	33.8	8.4
PC aa C42:0	0.7	0.2	0.6	0.2	0.6	0.1	0.6	0.2
PC aa C42:1	0.3	0.1	0.3	0.1	0.3	0.1	0.3	0.1
PC aa C42:2	0.2	0.1	0.2	0.1	0.2	0.1	0.3	0.1
PC aa C42:4	0.2	0.0	0.1	0.0	0.2	0.0	0.2	0.0
PC aa C42:5	0.4	0.1	0.3	0.1	0.4	0.2	0.4	0.1
PC aa C42:6	0.5	0.2	0.5	0.1	0.4	0.1	0.5	0.1
PC ae C30:0	0.4	0.1	0.4	0.1	0.4	0.1	0.4	0.1
PC ae C30:1	0.1	0.0	0.1	0.0	0.1	0.1	0.1	0.1
PC ae C30:2	0.1	0.0	0.1	0.0	0.1	0.0	0.1	0.1
PC ae C32:1	3.2	0.7	3.2	0.8	3.3	0.6	3.4	0.9
PC ae C32:2	0.8	0.2	0.9	0.3	0.8	0.2	0.8	0.2
PC ae C34:0	2.1	0.6	2.0	0.6	1.9	0.5	2.0	0.5
PC ae C34:1	11.9	2.2	12.4	3.1	12.5	2.8	12.6	2.6
PC ae C34:2	12.7	3.0	12.8	3.4	12.2	3.5	14.1	3.6
PC ae C34:3	8.0	1.8	7.9	2.5	7.8	2.7	9.2	2.9
PC ae C36:0	0.9	0.2	0.9	0.3	0.9	0.2	1.2	0.4
PC ae C36:1	10.1	2.0	10.3	2.7	10.1	2.1	10.3	1.8
PC ae C36:2	15.6	3.0	15.6	4.0	15.2	3.0	15.8	3.1
PC ae C36:3	7.3	1.4	7.6	2.1	7.4	1.8	8.7	2.1
PC ae C36:4	17.1	3.6	16.2	3.9	16.8	3.3	17.8	4.0
PC ae C36:5	12.7	2.7	11.1	2.8	12.1	3.3	13.3	3.6
PC ae C38:0	2.9	0.9	2.5	0.9	2.7	1.0	3.0	1.0
PC ae C38:1	0.3	0.3	0.6	0.5	0.4	0.4	1.5	2.2
PC ae C38:2	1.9	0.4	1.8	0.4	1.9	0.5	2.5	1.4
PC ae C38:3	4.4	0.8	4.6	1.0	4.6	1.0	4.5	0.9
PC ae C38:4	11.8	2.1	12.0	2.3	11.8	1.9	12.1	2.5
PC ae C38:5	15.2	2.7	14.7	2.9	15.7	2.7	16.5	3.0
PC ae C38:6	8.5	2.2	7.3	2.2	7.5	2.1	9.1	2.4
PC ae C40:1	1.4	0.4	1.3	0.3	1.4	0.4	1.6	0.4
PC ae C40:2	2.2	0.6	2.1	0.5	2.3	0.5	2.7	0.6
PC ae C40:3	1.0	0.2	1.0	0.2	1.0	0.1	1.2	0.3
PC ae C40:4	2.0	0.4	1.9	0.3	2.0	0.3	1.9	0.4
PC ae C40:5	3.4	0.6	3.4	0.5	3.4	0.5	3.7	0.8
PC ae C40:6	5.4	1.3	4.9	1.4	4.7	1.3	5.5	1.3
PC ae C42:1	0.3	0.1	0.3	0.1	0.4	0.1	0.3	0.1
PC ae C42:2	0.6	0.1	0.5	0.2	0.6	0.2	0.6	0.2
PC ae C42:3	0.7	0.2	0.8	0.1	0.8	0.2	0.9	0.2
PC ae C42:4	0.8	0.2	0.9	0.2	0.8	0.2	0.7	0.2
PC ae C42:5	1.7	0.4	1.7	0.3	1.7	0.2	1.8	0.4
PC ae C44:3	0.1	0.1	0.1	0.1	0.1	0.0	0.1	0.1
PC ae C44:4	0.3	0.1	0.4	0.1	0.4	0.1	0.4	0.2
PC ae C44:5	1.5	0.4	1.7	0.5	1.7	0.3	1.4	0.4
PC ae C44:6	1.1	0.3	1.1	0.3	1.1	0.2	1.1	0.4
SM (OH) C14:1	7.2	1.5	6.6	1.6	7.0	1.5	7.5	1.8
SM (OH) C16:1	3.9	0.8	3.6	0.9	3.7	0.8	3.9	0.9
SM (OH) C22:1	12.1	2.9	10.6	2.4	11.1	3.0	13.1	2.5
SM (OH) C22:2	11.8	2.6	10.9	2.9	10.8	2.3	12.5	2.8
SM (OH) C24:1	1.1	0.3	1.0	0.4	1.0	0.3	1.1	0.3
SM C16:0	123.1	21.0	117.4	27.4	134.3	29.1	131.6	25.7
SM C16:1	17.3	3.6	16.6	3.9	18.2	5.4	18.5	3.8
SM C18:0	25.3	4.8	24.5	6.4	26.1	7.1	25.6	5.9
SM C18:1	12.4	2.9	12.1	3.5	12.4	4.1	12.5	3.5
SM C20:2	0.3	0.1	0.2	0.1	0.3	0.1	0.3	0.2
SM C24:0	18.5	4.1	17.6	4.8	18.5	4.5	19.2	3.3
SM C24:1	55.7	11.5	54.8	16.0	59.4	12.5	58.8	11.8
SM C26:0	0.2	0.1	0.1	0.1	0.1	0.1	0.2	0.1
SM C26:1	0.4	0.2	0.4	0.1	0.4	0.2	0.4	0.1
Hexoses	5474.2	909.0	5406.3	1140.6	6180.5	1957.0	5410.4	539.4

ADMA, Asymmetric dimethylarginine; SDMA, Symmetric dimethylarginine; SM (OH): sphingolipid; lysoPC: lysophosphatidylcholine; PC: phosphatidylcholine.

Table S3. Summary of the statistics for the pair-wise OPLS-DA models used to describe group differences between the different disease and control groups.

Method	*Comparison*	*Variables*	A_{pred}	A_{orth}	R^2X	R^2Y	Q^2Y	*CV-ANOVA*	*AUC*
NMR	PD : Control	Selected	1	1	0.317	0.442	0.330	$7.9 \cdot 10^{-5}$	0.69 : 0.87
	MSA : Control	Selected	1	1	0.490	0.438	0.298	0.0080	0.74 : 0.81
	PSP : Control	All	1	1	0.160	0.791	0.472	$3.0 \cdot 10^{-5}$	0.86 : 0.80
	PD : MSA	Selected	1	0	0.351	0.256	0.221	0.0052	0.71 : 0.79
	PD : PSP	All	1	0	0.148	0.372	0.140	0.0359	0.70 : 0.84
	MSA : PSP	Selected	1	0	0.368	0.468	0.339	0.0085	0.64 : 0.77
MS	PD : Control	All	1	1	0.322	0.632	0.396	$4.5 \cdot 10^{-7}$	0.78 : 0.95
	MSA : Control	All	1	1	0.329	0.740	0.352	0.002	0.96 : 0.94
	PSP : Control	All	1	1	0.332	0.724	0.409	0.00025	0.89 : 0.85
	PD : MSA	Selected	1	0	0.282	0.396	0.277	0.0011	0.64 : 0.89
	PD : PSP	All	1	4	0.474	0.950	0.388	0.034	0.79 : 0.84
	MSA : PSP	Selected	1	1	0.442	0.735	0.571	0.00097	0.75 : 0.74

A_{pred}, number of predictive components; A_{orth}, number of orthogonal components; R^2X and R^2Y, explained variation in X and Y, respectively; Q^2Y, the quality and predictive power of the model. *CV-ANOVA* is a significance testing based on ANOVA of the cross-validated residuals. *AUC* is the area under curve in an ROC analysis and represents the ability to discriminate between groups.

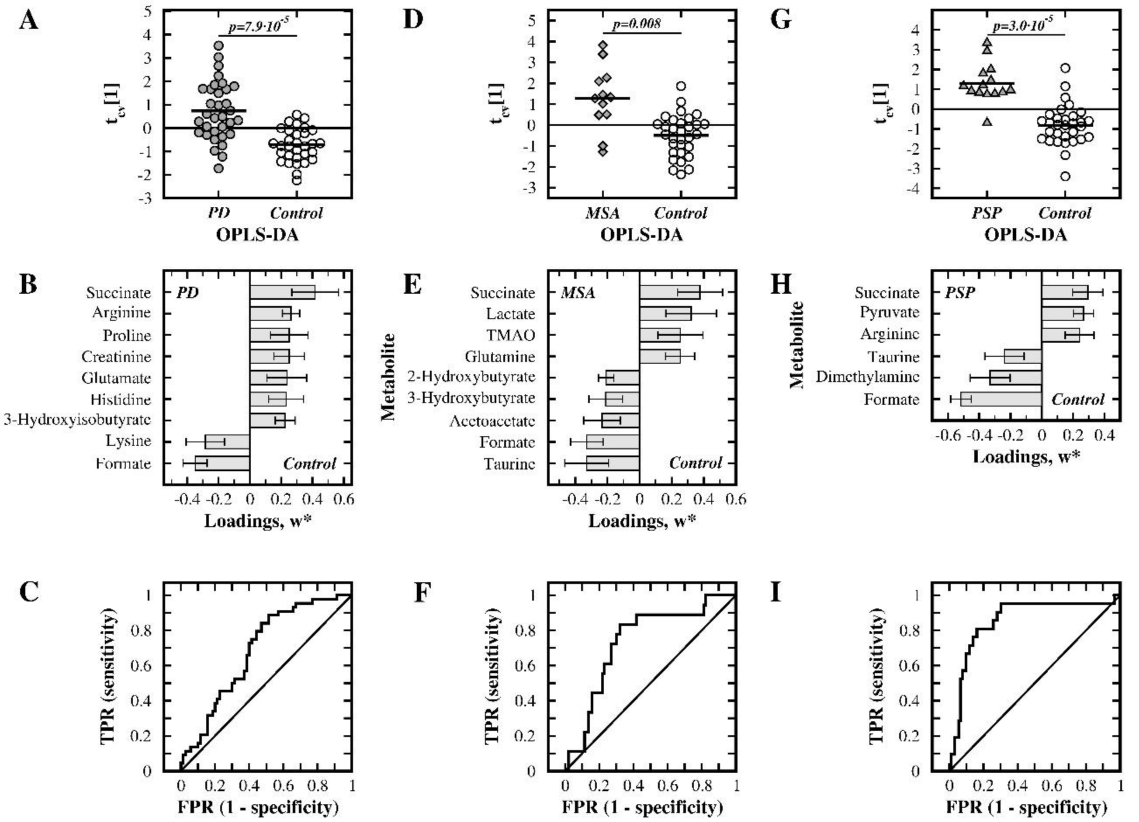

Figure S1:

Multivariate statistical comparisons between the patient groups and control group using the NMR data. Comparisons are shown in panels A–C) PD versus controls, D–F) MSA versus controls, and G–I) PSP versus controls. Cross-validated OPLS-DA score plots of the first component, $t_{cv}[1]$, are shown in panels A, D, and G. The most important metabolites in each model, as judged from their weight values (w*), are displayed in panels B, E, and H. ROC analyses for discriminating between groups are presented in panels C, F, and I (TPR: true positive rate, FPR: false positive rate). TMAO: trimethylamine N-oxide.

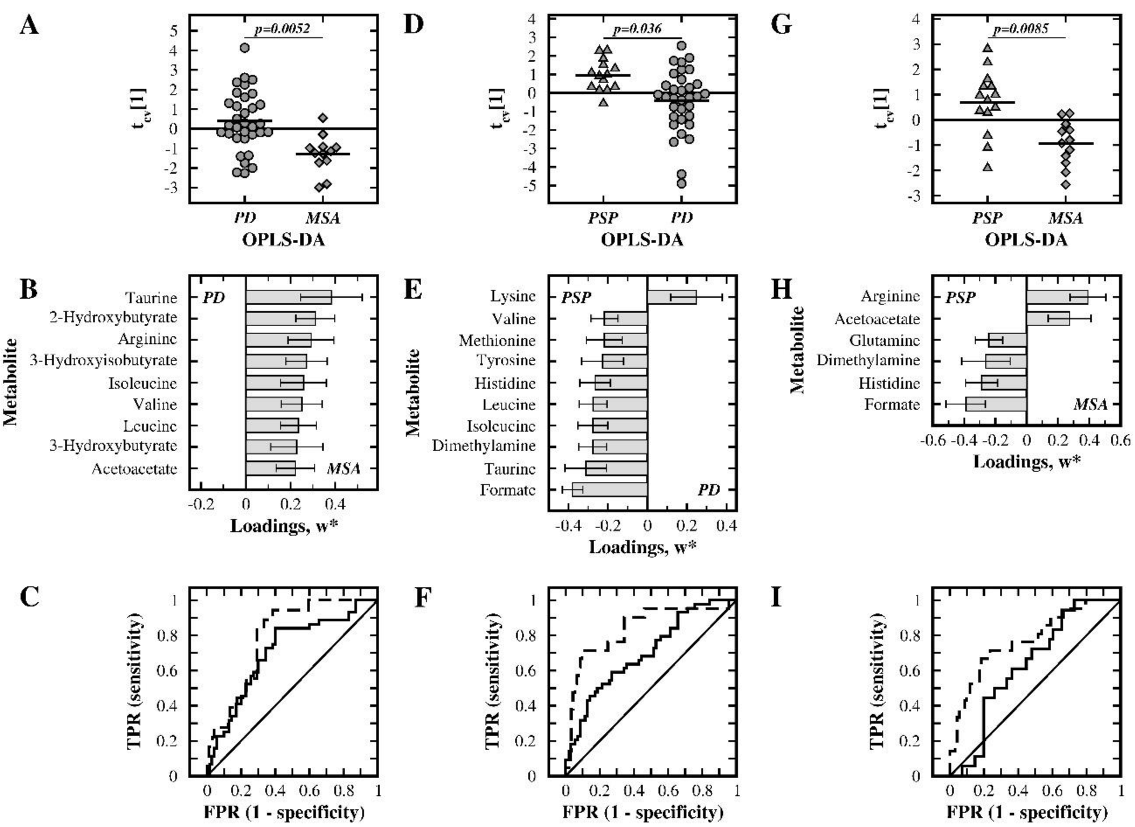

Figure S2:

Multivariate statistical comparisons between the patient groups using the NMR data. A–C) PD versus MSA, D–F) PD versus PSP, and G–I) MSA versus PSP. Cross-validated OPLS-DA score plots of the first component, $t_{cv}[1]$, are shown in panels A, D, and G. The most important metabolites in each model, as judged from their weight values (w*), are displayed in panels B, E, and H. ROC analyses for discriminating between groups are presented in panels C, F, and I (TPR: true positive rate, FPR: false positive rate).

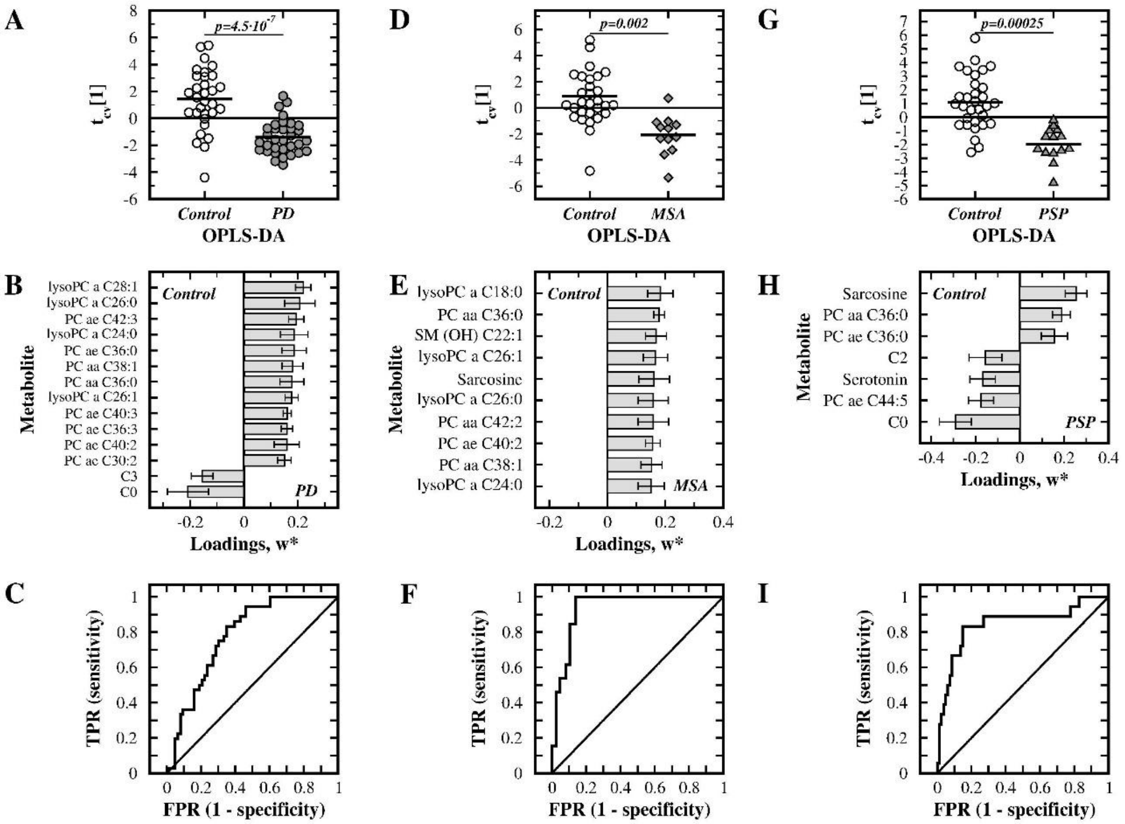

Figure S3:

Multivariate statistical comparisons between the patient groups and the control group using the MS data. Comparisons are shown in panels A–C) PD versus controls, D–F) MSA versus controls, and G–I) PSP versus controls. Cross-validated OPLS-DA score plots of the first component, $t_{cv}[1]$, are shown in panels A, D, and G. The most important metabolites in each model, as judged from their weight values (w*), are shown in panels B, E, and H. ROC analyses for discriminating between groups are presented in panels C, F, and I (TPR: true positive rate, FPR: false positive rate). C0: carnitine; C2: acetylcarnitine; lysoPC: lysophosphatidylcholine; PC: phosphatidylcholine; SM (OH): sphingolipid.

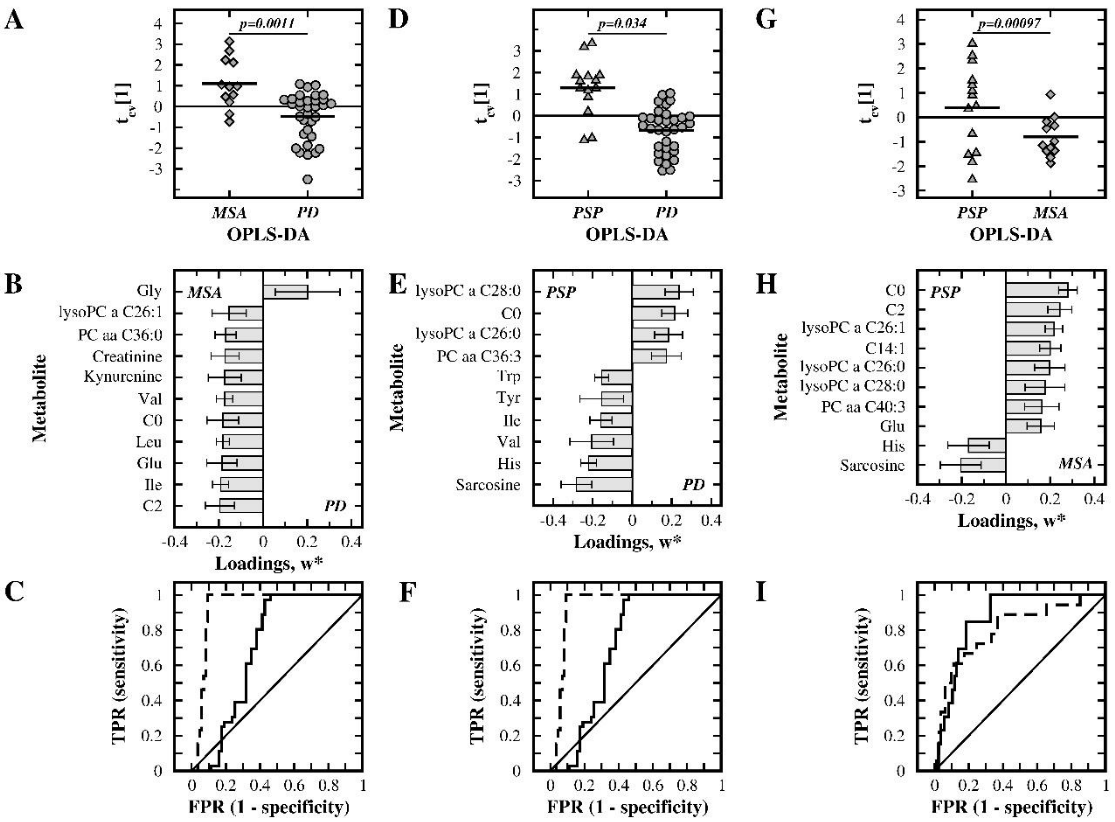

Figure S4:

Multivariate statistical comparisons between the patient groups using the MS data. A–C) PD versus MSA, D–F) PD versus PSP, and G–I) MSA versus PSP. Cross-validated OPLS-DA score plots of the first component, $t_{cv}[1]$, are shown in panels A, D, and G. The most important metabolites in each model, as judged from their weight values (|w*|), are shown in panels B, E, and H. ROC analyses for discriminating between groups are presented in panels C, F, and I (TPR: true positive rate, FPR: false positive rate). C0: carnitine; C2: acetylcarnitine; lysoPC: lysophosphatidylcholine; PC: phosphatidylcholine.

Table S4. Univariate and multivariate statistical analysis of NMR-detected metabolites in plasma. For each metabolite the fold change, the *p*-value from Student's t-tests, and weight values (w*) are listed.

**Metabolite**	**PD versus Control**			**MSA versus Control**			**PSP versus Control**			**PD versus MSA**			**PD versus PSP**			**MSA-p versus PSP-p**		
	fc	*p-value*	*w**	*fc*	*p-value*	*w**	*fc*	*p-value*	*w**	*fc*	*p-value*	*w**	*fc*	*p-value*	*w**	*fc*	*p-value*	*w**
2-Hydroxybutyrate	1.00	0.9693	-0.005	0.79	0.1229	-0.208	0.94	0.6907	-0.043	1.26	0.0270	0.311	1.06	0.6207	-0.062	0.84	0.2637	0.175
2-Hydroxyisobutyrate	1.29	0.4595	0.098	1.74	0.2314	0.162	2.54	0.0796	0.187	0.74	0.5261	-0.092	0.51	0.1721	0.171	0.68	0.5897	0.085
2-Hydroxyisovalerate	0.79	0.3161	-0.133	0.86	0.6190	-0.068	0.66	0.1847	-0.143	0.91	0.7897	-0.039	1.19	0.6267	-0.061	1.31	0.4733	-0.113
3-Hydroxybutyrate	1.02	0.9325	0.011	0.53	0.1182	-0.211	0.88	0.7058	-0.041	1.94	0.1100	0.228	1.17	0.6598	-0.056	0.60	0.2726	0.172
3-Hydroxyisobutyrate	1.14	0.0868	0.225	0.92	0.3720	-0.122	1.04	0.6793	0.045	1.23	0.0551	0.272	1.10	0.3793	-0.111	0.89	0.3026	0.162
Acetate	1.10	0.3197	0.132	0.97	0.7766	-0.039	0.93	0.5436	-0.066	1.14	0.2954	0.150	1.18	0.1601	-0.176	1.04	0.7105	-0.059
Acetoacetate	1.04	0.8584	0.024	0.58	0.0821	-0.234	0.94	0.8050	-0.027	1.81	0.1205	0.221	1.11	0.7285	-0.044	0.61	0.0748	0.274
Acetone	1.03	0.7205	0.047	0.93	0.4604	-0.101	1.15	0.4065	0.090	1.10	0.4090	0.119	0.90	0.5250	0.080	0.81	0.4328	0.124
Alanine	1.06	0.2187	0.162	1.03	0.5329	0.085	1.00	0.9989	0.000	1.03	0.6799	0.060	1.06	0.4183	-0.102	1.03	0.6934	-0.063
Arginine	1.34	0.0439	0.263	0.93	0.7200	-0.049	1.51	0.0223	0.241	1.45	0.0385	0.292	0.89	0.4091	0.104	0.61	0.0081	0.391
Citrate	1.01	0.9119	0.015	1.02	0.7596	0.042	1.01	0.8540	0.020	0.99	0.8728	-0.023	0.99	0.9382	0.010	1.01	0.9466	-0.011
Creatine	1.04	0.8994	0.017	0.78	0.2927	-0.143	1.06	0.7727	0.031	1.34	0.6228	0.071	0.98	0.9742	0.004	0.74	0.2726	0.172
Creatinine	1.13	0.0549	0.251	1.06	0.3427	0.129	1.18	0.1634	0.150	1.07	0.5071	0.096	0.96	0.7693	0.037	0.90	0.5705	0.090
Dimethylamine	0.96	0.5346	-0.082	0.90	0.1462	-0.196	0.76	0.0013	-0.330	1.07	0.4845	0.101	1.26	0.0245	-0.276	1.18	0.0911	-0.261
Formic acid	0.83	0.0066	-0.350	0.83	0.0128	-0.328	0.53	0.0000	-0.517	1.01	0.9432	0.010	1.57	0.0016	-0.378	1.56	0.0085	-0.389
Glucose	1.02	0.5810	0.073	0.99	0.8987	-0.017	1.11	0.0749	0.190	1.03	0.6615	0.063	0.92	0.2157	0.155	0.90	0.2418	0.183
Glutamate	1.17	0.0712	0.236	1.02	0.8492	0.026	1.15	0.2881	0.115	1.14	0.2381	0.169	1.02	0.8617	-0.022	0.89	0.4954	0.108
Glutamine	1.02	0.4660	0.097	1.07	0.0585	0.253	0.99	0.7838	-0.030	0.95	0.1740	-0.195	1.03	0.4315	-0.099	1.08	0.1187	-0.242
Histidine	1.07	0.0773	0.231	1.03	0.5180	0.088	0.96	0.2530	-0.123	1.04	0.4046	0.120	1.12	0.0320	-0.264	1.07	0.0600	-0.288
Isoleucine	1.08	0.2022	0.168	0.92	0.2836	-0.146	0.89	0.1243	-0.165	1.17	0.0690	0.258	1.22	0.0250	-0.276	1.04	0.5827	-0.087
Lactate	1.12	0.1614	0.184	1.24	0.0147	0.322	1.14	0.2476	0.125	0.90	0.3812	-0.126	0.99	0.9330	0.011	1.09	0.5541	-0.094
Leucine	1.06	0.3110	0.134	0.93	0.3174	-0.136	0.90	0.1075	-0.172	1.13	0.0979	0.236	1.18	0.0257	-0.274	1.04	0.4992	-0.107
Lysine	0.74	0.0286	-0.285	0.88	0.4329	-0.107	1.03	0.8096	0.026	0.84	0.3802	-0.126	0.72	0.0451	0.248	0.86	0.3141	0.158
Mannose	1.03	0.7961	0.034	1.02	0.9143	0.015	1.23	0.1643	0.149	1.02	0.9268	0.013	0.84	0.2709	0.138	0.83	0.2900	0.166
Methionine	1.10	0.3392	0.126	1.01	0.9571	0.007	0.90	0.4204	-0.087	1.09	0.4477	0.109	1.22	0.0794	-0.218	1.12	0.2865	-0.167
Phenylalanine	1.05	0.2580	0.149	0.98	0.7078	-0.051	1.01	0.7758	0.031	1.07	0.2935	0.151	1.04	0.5540	-0.075	0.97	0.5940	0.084
Proline	1.12	0.0545	0.251	1.03	0.6924	0.054	1.05	0.5197	0.070	1.08	0.2887	0.153	1.06	0.4168	-0.102	0.98	0.8390	0.032
Pyruvate	1.09	0.2376	0.156	1.07	0.4005	0.115	1.23	0.0109	0.267	1.01	0.8939	0.019	0.89	0.1989	0.161	0.88	0.2107	0.196
Succinate	1.23	0.0010	0.417	1.21	0.0038	0.377	1.21	0.0046	0.294	1.02	0.8053	0.036	1.02	0.7905	-0.034	1.00	0.9937	-0.001
Taurine	1.01	0.8114	0.032	0.81	0.0123	-0.330	0.85	0.0237	-0.239	1.26	0.0056	0.383	1.19	0.0106	-0.312	0.94	0.6304	0.076
Threonine	1.03	0.4523	0.100	1.02	0.6718	0.058	0.94	0.2413	-0.126	1.00	0.9399	0.011	1.10	0.1310	-0.189	1.09	0.3398	-0.150
Trimethylamine N-oxide	1.03	0.6543	0.059	1.19	0.0577	0.254	1.10	0.2039	0.137	0.87	0.1849	-0.190	0.93	0.4772	0.090	1.08	0.5731	-0.089
Tyrosine	1.01	0.8246	0.029	0.93	0.3820	-0.119	0.88	0.1114	-0.171	1.09	0.2852	0.154	1.15	0.0681	-0.226	1.05	0.5864	-0.086
Valine	1.04	0.3966	0.112	0.93	0.2341	-0.161	0.93	0.2447	-0.125	1.12	0.0781	0.250	1.12	0.0807	-0.217	1.00	0.9932	-0.001

Table S5. Univariate and multivariate statistical analysis of MS-detected metabolites in plasma. For each metabolite the fold change, the *p*-value from Student's t-tests, and the weight values (w*) are listed.

Metabolite	PD versus Control			MSA-p versus Control			PSP-p versus Control			PD versus MSA-p			PD versus PSP-p			MSA-p versus PSP-p		
	fc	*p-value*	*w**	*fc*	*p-value*	*w**	*fc*	*p-value*	*w**	*fc*	*p-value*	*w**	*fc*	*p-value*	*w**	*fc*	*p-value*	*w**
Alanine	1.05	0.35735	-0.045	1.03	0.66732	-0.024	1.10	0.19420	-0.079	1.02	0.81638	-0.017	0.96	0.51383	0.058	0.94	0.52010	0.059
Arginine	0.97	0.55403	0.037	0.87	0.05834	0.115	0.99	0.93909	0.007	1.11	0.19993	-0.109	0.97	0.71548	0.032	0.88	0.14319	0.132
Asparagine	0.99	0.84719	0.015	0.91	0.20141	0.082	0.94	0.34709	0.062	1.09	0.24476	-0.104	1.06	0.41855	-0.067	0.97	0.70625	0.040
Citrulline	1.01	0.88058	-0.003	0.89	0.18753	0.082	1.03	0.66315	-0.024	1.14	0.20153	-0.110	0.98	0.77946	0.026	0.86	0.16797	0.126
Glutamine	1.03	0.32465	-0.045	1.09	0.05455	-0.107	1.04	0.39968	-0.048	0.94	0.12748	0.134	0.99	0.85868	0.020	1.06	0.33100	-0.088
Glutamate	1.19	0.09502	-0.087	0.86	0.34109	0.058	1.18	0.24451	-0.069	1.38	0.02802	-0.186	1.01	0.92189	-0.007	0.73	0.07967	0.157
Glycine	0.87	0.08497	0.097	1.11	0.37472	-0.051	0.92	0.42570	0.052	0.78	0.01634	0.202	0.94	0.48013	0.061	1.21	0.11650	-0.140
Histidine	1.05	0.10385	-0.084	1.02	0.53196	-0.035	0.92	0.01903	0.146	1.03	0.56075	-0.047	1.15	0.00625	-0.220	1.11	0.05782	-0.169
Isoleucine	1.10	0.11285	-0.083	0.90	0.12491	0.094	0.94	0.34286	0.062	1.23	0.02211	-0.192	1.17	0.05293	-0.158	0.95	0.49702	0.063
Leucine	1.04	0.45951	-0.036	0.88	0.05756	0.114	0.91	0.16514	0.089	1.19	0.02977	-0.182	1.14	0.07486	-0.146	0.96	0.53872	0.057
Lysine	1.10	0.02349	-0.115	1.14	0.02131	-0.130	1.10	0.08528	-0.100	0.96	0.48241	0.071	1.00	0.95627	-0.001	1.04	0.51072	-0.065
Methionine	0.96	0.53241	0.038	0.94	0.37827	0.057	0.87	0.03424	0.133	1.03	0.79486	-0.024	1.11	0.25941	-0.094	1.08	0.38041	-0.079
Ornitine	1.16	0.01444	-0.127	1.13	0.06502	-0.102	1.16	0.01999	-0.133	1.02	0.76049	-0.022	1.00	0.99449	0.005	0.98	0.69379	0.035
Phenylalanine	1.03	0.42621	-0.040	0.96	0.38212	0.058	1.00	0.96308	0.005	1.08	0.16932	-0.121	1.04	0.53081	-0.051	0.96	0.50389	0.066
Proline	1.05	0.37938	-0.047	0.98	0.84732	0.016	1.07	0.45065	-0.046	1.07	0.40397	-0.076	0.99	0.88154	0.013	0.92	0.48705	0.067
Serine	1.02	0.67096	-0.018	0.99	0.90505	0.010	1.02	0.76858	-0.016	1.03	0.69376	-0.032	1.00	0.97090	-0.001	0.97	0.77245	0.027
Threonine	1.01	0.81987	-0.008	1.02	0.71880	-0.014	0.97	0.54960	0.040	0.99	0.87348	0.009	1.05	0.53579	-0.051	1.06	0.57840	-0.046
Tryptophan	1.02	0.62991	-0.017	0.90	0.04916	0.118	0.90	0.03640	0.130	1.13	0.08278	-0.148	1.14	0.05951	-0.154	1.00	0.95268	-0.004
Tyrosine	1.02	0.76295	-0.012	0.89	0.17471	0.084	0.87	0.09765	0.104	1.14	0.11328	-0.135	1.17	0.05920	-0.154	1.03	0.78854	-0.023
Valine	1.05	0.23773	-0.061	0.94	0.20791	0.081	0.90	0.07231	0.114	1.12	0.04375	-0.174	1.16	0.01195	-0.204	1.03	0.56398	-0.048
ADMA	0.99	0.87862	0.010	0.93	0.18101	0.076	1.00	0.93182	0.009	1.07	0.32986	-0.079	1.00	0.96909	0.001	0.94	0.35976	0.077
Creatinine	1.13	0.08607	-0.096	0.90	0.17283	0.085	1.07	0.52000	-0.040	1.25	0.04725	-0.172	1.05	0.70689	-0.034	0.84	0.33454	0.089
Kynurenine	1.13	0.09700	-0.088	0.90	0.26501	0.071	1.19	0.14081	-0.091	1.25	0.04339	-0.173	0.95	0.66422	0.038	0.76	0.13313	0.137
Sarcosine	0.91	0.11061	0.090	0.78	0.00579	0.161	0.66	0.00001	0.254	1.15	0.09484	-0.145	1.38	0.00036	-0.282	1.19	0.01876	-0.204
SDMA	1.12	0.03729	-0.112	1.11	0.18257	-0.078	1.23	0.02587	-0.136	1.01	0.93118	-0.008	0.91	0.31349	0.085	0.90	0.49477	0.063
Serotonin	1.78	0.00656	-0.146	1.07	0.78333	-0.015	2.11	0.00518	-0.167	1.66	0.12108	-0.133	0.84	0.50921	0.056	0.51	0.10443	0.146
Taurine	–	–	–	–	–	–	–	–	–	1.03	0.76482	-0.026	0.96	0.65771	0.038	0.93	0.57313	0.052
Carnitine	1.28	0.00007	-0.208	1.06	0.42032	-0.049	1.56	0.00000	-0.291	1.20	0.03168	-0.182	0.82	0.00786	0.216	0.68	0.00058	0.281
Acetylcarnitine	1.17	0.09223	-0.091	0.87	0.27597	0.064	1.36	0.01003	-0.155	1.35	0.01889	-0.195	0.86	0.15915	0.118	0.64	0.00369	0.244
Propionylcarnitine	1.20	0.00400	-0.155	1.12	0.14340	-0.088	1.17	0.03208	-0.131	1.07	0.40185	-0.071	1.03	0.75734	-0.027	0.95	0.61097	0.044
Malonylcarnitine	1.19	0.48017	-0.038	0.99	0.94225	0.006	1.23	0.15946	-0.086	1.21	0.62595	-0.042	0.96	0.90930	0.010	0.80	0.16569	0.125
Tetradecenoylcarnitine	1.13	0.24449	-0.062	0.88	0.33386	0.061	1.31	0.02645	-0.136	1.29	0.12946	-0.131	0.87	0.29679	0.090	0.67	0.02367	0.200
lysoPC a C16:0	0.95	0.26164	0.069	0.87	0.03122	0.126	0.99	0.86992	0.014	1.10	0.18024	-0.112	0.96	0.50875	0.060	0.88	0.12967	0.137
lysoPC a C16:1	1.00	0.96771	0.003	1.02	0.87323	-0.005	1.09	0.36445	-0.053	0.98	0.87486	0.012	0.92	0.29311	0.090	0.93	0.55013	0.058
lysoPC a C17:0	1.00	0.99615	0.003	0.92	0.31987	0.063	0.93	0.36718	0.058	1.08	0.33468	-0.087	1.07	0.37640	-0.074	0.99	0.95442	0.009
lysoPC a C18:0	0.93	0.13549	0.088	0.82	0.00122	0.183	0.94	0.29191	0.069	1.14	0.08829	-0.143	0.99	0.93142	0.010	0.87	0.14817	0.130
lysoPC a C18:1	0.92	0.18086	0.081	0.94	0.50646	0.042	1.00	0.97576	0.001	0.98	0.76888	0.032	0.92	0.19922	0.114	0.94	0.46151	0.067
lysoPC a C18:2	0.94	0.47214	0.043	0.86	0.25116	0.070	0.92	0.50613	0.043	1.10	0.41256	-0.069	1.02	0.87116	-0.011	0.93	0.56062	0.054
lysoPC a C20:3	0.93	0.28744	0.061	0.96	0.66905	0.026	1.05	0.57879	-0.035	0.97	0.74588	0.030	0.88	0.19219	0.112	0.91	0.47885	0.066
lysoPC a C20:4	0.98	0.83637	0.017	0.89	0.31833	0.061	1.04	0.69008	-0.022	1.10	0.44012	-0.064	0.95	0.59135	0.048	0.86	0.15245	0.128

lysoPC a C24:0	0.68	0.00050	0.188	0.64	0.00652	0.151	0.77	0.06456	0.111	1.06	0.67640	-0.013	0.89	0.37226	0.089	0.84	0.28066	0.094
lysoPC a C26:0	0.52	0.00006	0.208	0.51	0.00495	0.158	0.73	0.09916	0.099	1.03	0.87725	-0.008	0.71	0.03105	0.184	0.70	0.02451	0.198
lysoPC a C26:1	0.63	0.00097	0.178	0.50	0.00384	0.166	0.70	0.06098	0.111	1.28	0.07683	-0.153	0.90	0.37185	0.094	0.71	0.01820	0.218
lysoPC a C28:0	0.76	0.00767	0.150	0.80	0.12987	0.090	1.00	0.98273	-0.001	0.95	0.60779	0.055	0.76	0.00499	0.238	0.80	0.04622	0.176
lysoPC a C28:1	0.59	0.00002	0.221	0.62	0.01218	0.143	0.69	0.02559	0.136	0.95	0.58306	0.060	0.85	0.10003	0.144	0.90	0.40758	0.072
PC aa C24:0	0.81	0.09472	0.095	0.80	0.21810	0.072	1.02	0.90693	-0.008	1.01	0.95906	0.004	0.79	0.10196	0.141	0.79	0.14798	0.128
PC aa C28:1	0.94	0.27426	0.063	0.84	0.05523	0.113	0.99	0.86887	0.011	1.11	0.20997	-0.106	0.95	0.47686	0.062	0.85	0.10973	0.144
PC aa C30:0	1.02	0.79330	-0.010	0.89	0.28524	0.068	0.99	0.93474	0.007	1.15	0.23935	-0.103	1.03	0.78748	-0.021	0.90	0.42312	0.076
PC aa C30:2	1.15	0.51598	-0.046	1.21	0.38859	-0.076	1.11	0.67503	-0.029	0.95	0.83574	0.037	1.04	0.88919	-0.021	1.09	0.66754	-0.073
PC aa C32:0	0.98	0.73525	0.025	0.93	0.34117	0.057	1.06	0.40372	-0.050	1.06	0.46613	-0.057	0.92	0.27327	0.095	0.87	0.18868	0.118
PC aa C32:1	0.93	0.52179	0.038	1.04	0.78615	-0.014	1.09	0.57597	-0.034	0.90	0.45407	0.064	0.86	0.28540	0.091	0.96	0.84292	0.020
PC aa C32:2	0.89	0.20729	0.070	0.82	0.14570	0.090	0.93	0.56364	0.037	1.08	0.56860	-0.054	0.96	0.73453	0.029	0.89	0.48416	0.068
PC aa C32:3	0.99	0.85832	0.011	0.92	0.34931	0.053	1.01	0.94180	-0.004	1.08	0.43901	-0.063	0.98	0.84475	0.017	0.91	0.47372	0.063
PC aa C34:1	0.97	0.60815	0.033	1.06	0.50113	-0.041	1.10	0.23304	-0.073	0.92	0.32688	0.091	0.89	0.13182	0.128	0.96	0.76114	0.025
PC aa C34:2	0.94	0.16029	0.082	0.94	0.29845	0.063	1.02	0.75569	-0.019	1.00	0.97221	0.001	0.92	0.21514	0.107	0.92	0.36883	0.082
PC aa C34:3	0.90	0.14249	0.082	0.90	0.32947	0.058	1.03	0.79598	-0.016	0.99	0.95080	0.008	0.88	0.18291	0.112	0.88	0.35790	0.083
PC aa C34:4	0.95	0.55509	0.034	0.89	0.35551	0.055	0.97	0.80716	0.017	1.06	0.66401	-0.036	0.98	0.85917	0.015	0.92	0.63703	0.042
PC aa C36:0	0.74	0.00096	0.179	0.61	0.00216	0.179	0.63	0.00181	0.188	1.22	0.05749	-0.169	1.17	0.09159	-0.139	0.96	0.76412	0.035
PC aa C36:1	0.97	0.56984	0.035	1.05	0.60972	-0.037	1.03	0.68811	-0.024	0.92	0.42922	0.079	0.94	0.47293	0.062	1.01	0.92998	-0.014
PC aa C36:2	0.92	0.10596	0.092	0.89	0.07236	0.107	0.95	0.43488	0.049	1.03	0.68071	-0.033	0.97	0.66117	0.040	0.94	0.46660	0.067
PC aa C36:3	0.93	0.15637	0.083	0.99	0.89702	0.009	1.08	0.26523	-0.067	0.94	0.38175	0.079	0.86	0.03842	0.173	0.92	0.37957	0.080
PC aa C36:4	0.97	0.59487	0.032	0.92	0.30739	0.061	1.07	0.39596	-0.053	1.05	0.64122	-0.038	0.90	0.27448	0.094	0.86	0.18495	0.120
PC aa C36:5	0.78	0.06428	0.103	0.65	0.05254	0.114	0.74	0.11970	0.097	1.20	0.25843	-0.095	1.06	0.68925	-0.032	0.89	0.53973	0.056
PC aa C36:6	0.85	0.11571	0.087	0.73	0.03631	0.121	0.79	0.09670	0.103	1.17	0.26825	-0.093	1.08	0.56810	-0.048	0.92	0.65141	0.040
PC aa C38:0	0.93	0.27873	0.065	0.86	0.14714	0.086	0.83	0.03783	0.128	1.08	0.44269	-0.061	1.13	0.17981	-0.108	1.04	0.73665	-0.032
PC aa C38:1	0.59	0.00080	0.181	0.61	0.01385	0.153	0.69	0.05112	0.123	0.96	0.85440	0.004	0.85	0.45723	0.066	0.88	0.57427	0.069
PC aa C38:3	0.97	0.62085	0.032	0.99	0.87885	0.008	1.10	0.23764	-0.072	0.98	0.84151	0.023	0.88	0.14577	0.124	0.90	0.36681	0.080
PC aa C38:4	0.97	0.67310	0.027	0.93	0.36937	0.053	1.06	0.50908	-0.039	1.05	0.64376	-0.036	0.92	0.38443	0.075	0.88	0.28041	0.096
PC aa C38:5	0.92	0.22835	0.070	0.86	0.10624	0.093	0.93	0.42506	0.052	1.07	0.45444	-0.057	0.99	0.91742	0.011	0.92	0.48668	0.059
PC aa C38:6	0.98	0.80071	0.018	0.83	0.04573	0.116	0.91	0.31838	0.063	1.18	0.10708	-0.133	1.08	0.45168	-0.060	0.91	0.53664	0.055
PC aa C40:2	0.80	0.03765	0.115	0.77	0.08213	0.101	0.78	0.09376	0.103	1.05	0.64740	-0.030	1.03	0.78570	-0.016	0.98	0.86181	0.014
PC aa C40:3	0.82	0.01424	0.134	0.76	0.01349	0.145	0.88	0.19514	0.082	1.09	0.38388	-0.076	0.93	0.42514	0.069	0.86	0.07240	0.163
PC aa C40:4	1.01	0.78011	-0.009	1.09	0.29650	-0.059	1.13	0.23599	-0.072	0.93	0.42734	0.071	0.90	0.30988	0.088	0.96	0.82222	0.022
PC aa C40:5	1.00	0.97713	0.002	0.97	0.70947	0.019	1.01	0.92482	-0.006	1.04	0.72338	-0.023	0.99	0.94550	0.009	0.96	0.75977	0.025
PC aa C40:6	1.01	0.93069	0.000	0.85	0.09782	0.097	0.91	0.35458	0.060	1.18	0.11911	-0.129	1.10	0.35746	-0.075	0.93	0.64258	0.041
PC aa C42:0	1.04	0.63765	-0.019	1.01	0.95451	-0.004	0.95	0.59327	0.034	1.03	0.77071	-0.017	1.10	0.34489	-0.071	1.06	0.59156	-0.050
PC aa C42:1	0.91	0.33280	0.059	0.93	0.61076	0.027	0.93	0.53871	0.041	0.98	0.84864	0.029	0.98	0.84821	0.021	1.00	0.97733	-0.012
PC aa C42:2	0.84	0.01392	0.133	0.75	0.00961	0.158	0.78	0.02157	0.142	1.12	0.21375	-0.126	1.08	0.42649	-0.069	0.96	0.75746	0.042
PC aa C42:4	1.00	0.98759	0.003	0.95	0.58083	0.038	0.96	0.69163	0.025	1.06	0.58453	-0.048	1.04	0.68999	-0.028	0.98	0.87650	0.021
PC aa C42:5	0.94	0.40486	0.051	0.85	0.15864	0.090	0.93	0.53073	0.041	1.10	0.31814	-0.089	1.01	0.93899	-0.001	0.92	0.52247	0.067
PC aa C42:6	0.92	0.29338	0.062	0.89	0.25216	0.068	0.87	0.15588	0.089	1.03	0.80034	-0.017	1.06	0.62048	-0.037	1.03	0.80647	-0.024
PC ae C30:0	0.92	0.27382	0.062	0.90	0.33601	0.059	0.88	0.26655	0.070	1.02	0.83395	-0.018	1.04	0.71659	-0.030	1.02	0.90301	-0.010
PC ae C30:1	0.94	0.72682	0.019	0.89	0.67495	0.022	1.12	0.64032	-0.035	1.06	0.69907	-0.025	0.84	0.21804	0.112	0.79	0.26333	0.106
PC ae C30:2	0.75	0.00488	0.151	0.70	0.03332	0.126	0.78	0.09431	0.101	1.06	0.53523	-0.061	0.96	0.63546	0.045	0.90	0.37752	0.090
PC ae C32:1	0.94	0.34115	0.052	0.95	0.59504	0.029	0.98	0.81133	0.012	0.99	0.88321	0.017	0.96	0.54649	0.054	0.97	0.72266	0.031
PC ae C32:2	1.00	0.97793	0.007	1.03	0.74032	-0.018	0.97	0.72846	0.021	0.97	0.70697	0.035	1.03	0.72359	-0.022	1.06	0.58312	-0.047
PC ae C34:0	1.05	0.46997	-0.036	0.97	0.74317	0.015	0.97	0.69124	0.024	1.08	0.45608	-0.055	1.08	0.39959	-0.065	1.00	0.97789	-0.007
PC ae C34:1	0.94	0.24418	0.069	0.99	0.89842	0.008	0.99	0.90120	0.010	0.95	0.48099	0.066	0.95	0.43816	0.068	1.00	0.99193	-0.001

PC ae C34:2	0.90	0.10541	0.092	0.91	0.29146	0.063	0.87	0.10822	0.100	1.00	0.95391	0.009	1.04	0.59714	-0.041	1.05	0.66700	-0.040
PC ae C34:3	0.87	0.04990	0.113	0.86	0.18603	0.082	0.85	0.14743	0.091	1.01	0.89715	-0.011	1.02	0.81830	-0.014	1.01	0.94939	-0.003
PC ae C36:0	0.77	0.00054	0.187	0.76	0.02497	0.129	0.76	0.01054	0.156	1.02	0.84354	-0.004	1.02	0.79918	-0.014	1.00	0.99736	-0.006
PC ae C36:1	0.98	0.70820	0.025	1.00	0.97919	0.004	0.98	0.79012	0.017	0.98	0.83202	0.019	1.00	0.98324	0.006	1.01	0.88154	-0.011
PC ae C36:2	0.99	0.78725	0.019	0.99	0.85414	0.010	0.96	0.57157	0.036	1.00	0.99587	0.006	1.02	0.70814	-0.027	1.02	0.80014	-0.025
PC ae C36:3	0.84	0.00356	0.160	0.88	0.14987	0.085	0.85	0.05019	0.121	0.96	0.58964	0.053	1.00	0.94221	0.011	1.04	0.73845	-0.032
PC ae C36:4	0.96	0.49018	0.044	0.91	0.25958	0.067	0.95	0.43492	0.050	1.05	0.47929	-0.056	1.02	0.78823	-0.018	0.97	0.68096	0.036
PC ae C36:5	0.96	0.48188	0.045	0.83	0.06266	0.109	0.91	0.28230	0.069	1.15	0.08158	-0.142	1.06	0.47401	-0.056	0.92	0.42862	0.070
PC ae C38:0	0.97	0.69397	0.024	0.84	0.14233	0.088	0.91	0.36588	0.057	1.15	0.19339	-0.109	1.07	0.50366	-0.053	0.93	0.59687	0.049
PC ae C38:1	0.24	0.00514	0.149	0.37	0.16448	0.083	0.26	0.07384	0.111	0.63	0.10135	0.130	0.92	0.76821	0.016	1.47	0.33422	-0.086
PC ae C38:2	0.77	0.02491	0.126	0.72	0.09280	0.099	0.75	0.11173	0.101	1.07	0.41689	-0.057	1.02	0.79190	-0.018	0.96	0.66858	0.032
PC ae C38:3	0.98	0.66306	0.031	1.02	0.75759	-0.013	1.03	0.60564	-0.029	0.96	0.53333	0.055	0.95	0.39064	0.076	0.99	0.89274	0.015
PC ae C38:4	0.98	0.66396	0.030	1.00	0.94795	0.001	0.98	0.77203	0.020	0.98	0.78787	0.035	1.00	0.96341	0.010	1.01	0.84464	-0.024
PC ae C38:5	0.92	0.06665	0.108	0.89	0.07174	0.104	0.95	0.39403	0.055	1.04	0.56836	-0.039	0.97	0.54672	0.058	0.93	0.34354	0.083
PC ae C38:6	0.93	0.26906	0.064	0.81	0.03396	0.125	0.82	0.03647	0.130	1.15	0.13393	-0.126	1.13	0.15975	-0.116	0.98	0.87541	0.014
PC ae C40:1	0.92	0.26985	0.063	0.82	0.04501	0.116	0.89	0.21542	0.077	1.12	0.27149	-0.089	1.03	0.73016	-0.025	0.92	0.46481	0.066
PC ae C40:2	0.83	0.00360	0.160	0.79	0.00578	0.157	0.85	0.04174	0.126	1.06	0.49360	-0.052	0.98	0.75749	0.030	0.92	0.35136	0.081
PC ae C40:3	0.85	0.00426	0.161	0.85	0.05138	0.117	0.88	0.08498	0.109	1.00	0.96208	0.012	0.96	0.50804	0.065	0.96	0.59366	0.050
PC ae C40:4	1.02	0.75840	-0.016	0.99	0.83658	0.013	1.02	0.68454	-0.023	1.03	0.65906	-0.037	0.99	0.87884	0.012	0.96	0.55656	0.052
PC ae C40:5	0.93	0.12106	0.093	0.92	0.20299	0.083	0.91	0.14407	0.093	1.01	0.82075	-0.020	1.02	0.72299	-0.022	1.01	0.92197	0.000
PC ae C40:6	0.98	0.69325	0.025	0.88	0.15008	0.085	0.86	0.06591	0.114	1.11	0.25522	-0.095	1.14	0.13013	-0.124	1.03	0.81274	-0.022
PC ae C42:1	0.99	0.86159	0.018	1.01	0.93443	-0.009	1.09	0.35110	-0.058	0.98	0.80778	0.038	0.90	0.25219	0.102	0.92	0.50837	0.056
PC ae C42:2	0.91	0.18333	0.078	0.86	0.12496	0.096	0.99	0.88855	0.011	1.07	0.46805	-0.065	0.93	0.36902	0.078	0.87	0.24038	0.111
PC ae C42:3	0.78	0.00029	0.194	0.81	0.01659	0.141	0.87	0.10874	0.101	0.97	0.69931	0.037	0.90	0.21026	0.109	0.93	0.44722	0.072
PC ae C42:4	1.12	0.04153	-0.109	1.21	0.02563	-0.135	1.15	0.06515	-0.118	0.93	0.27010	0.106	0.97	0.63720	0.048	1.05	0.61792	-0.049
PC ae C42:5	0.96	0.44740	0.049	0.97	0.70153	0.028	0.97	0.62185	0.033	0.99	0.84706	0.018	0.99	0.87298	0.020	1.00	0.95577	0.001
PC ae C44:3	1.09	0.43344	-0.043	1.03	0.81127	-0.016	1.07	0.55040	-0.036	1.05	0.73462	-0.026	1.01	0.92138	-0.010	0.96	0.78559	0.020
PC ae C44:4	0.97	0.78617	0.018	1.09	0.51345	-0.043	1.09	0.50899	-0.042	0.89	0.21166	0.119	0.90	0.20521	0.113	1.00	0.95733	-0.013
PC ae C44:5	1.12	0.10430	-0.087	1.21	0.04424	-0.118	1.27	0.00359	-0.175	0.92	0.35043	0.082	0.88	0.08558	0.146	0.95	0.62721	0.047
PC ae C44:6	1.03	0.70566	-0.015	1.06	0.61210	-0.030	1.01	0.88206	-0.008	0.98	0.79677	0.028	1.02	0.86107	-0.009	1.04	0.68070	-0.039
SM (OH) C14:1	0.96	0.47416	0.043	0.88	0.13446	0.089	0.93	0.36212	0.059	1.09	0.24986	-0.095	1.03	0.66889	-0.034	0.94	0.51879	0.057
SM (OH) C16:1	0.99	0.85961	0.015	0.93	0.40393	0.053	0.96	0.57024	0.039	1.06	0.41290	-0.071	1.03	0.61560	-0.041	0.97	0.77203	0.028
SM (OH) C22:1	0.92	0.12110	0.092	0.81	0.00417	0.168	0.85	0.02587	0.140	1.14	0.11253	-0.134	1.08	0.32652	-0.079	0.95	0.58809	0.051
SM (OH) C22:2	0.95	0.35445	0.055	0.87	0.10574	0.099	0.87	0.06128	0.117	1.09	0.29403	-0.092	1.09	0.21249	-0.102	1.01	0.95643	-0.001
SM (OH) C24:1	0.97	0.64640	0.026	0.89	0.26661	0.070	0.87	0.11693	0.100	1.08	0.48407	-0.065	1.11	0.28881	-0.090	1.03	0.82107	-0.018
SM C16:0	0.93	0.14889	0.085	0.89	0.11746	0.095	1.02	0.75938	-0.017	1.05	0.46572	-0.060	0.92	0.14417	0.125	0.87	0.14300	0.133
SM C16:1	0.94	0.22956	0.075	0.90	0.15701	0.090	0.98	0.84090	0.015	1.04	0.55430	-0.052	0.95	0.53177	0.058	0.91	0.40228	0.081
SM C18:0	0.99	0.85086	0.017	0.96	0.60081	0.037	1.02	0.80836	-0.012	1.03	0.63917	-0.042	0.97	0.67376	0.038	0.94	0.55877	0.057
SM C18:1	0.99	0.90203	0.014	0.96	0.71161	0.026	0.99	0.88509	0.012	1.03	0.74169	-0.026	1.01	0.94411	-0.002	0.98	0.86171	0.017
SM C20:2	0.85	0.21688	0.072	0.78	0.26696	0.067	0.89	0.51724	0.039	1.08	0.56757	-0.044	0.96	0.68740	0.046	0.88	0.43251	0.078
SM C24:0	0.96	0.46145	0.047	0.91	0.20766	0.074	0.96	0.53789	0.042	1.05	0.51268	-0.049	1.00	0.97006	0.001	0.95	0.62587	0.041
SM C24:1	0.95	0.28112	0.065	0.93	0.36634	0.059	1.01	0.88508	-0.006	1.02	0.83630	-0.020	0.94	0.32616	0.086	0.92	0.41650	0.078
SM C26:0	0.75	0.01718	0.129	0.64	0.02574	0.131	0.67	0.02879	0.132	1.16	0.31103	-0.089	1.12	0.42914	-0.061	0.97	0.84417	0.025
SM C26:1	0.98	0.86646	0.015	0.88	0.24875	0.071	1.02	0.86031	-0.008	1.13	0.37633	-0.074	0.97	0.78047	0.026	0.86	0.30320	0.093
Hexoses	1.01	0.73590	-0.009	1.00	0.98721	0.004	1.14	0.04648	-0.120	1.01	0.83698	-0.012	0.89	0.09578	0.142	0.87	0.24025	0.106

ADMA, Asymmetric dimethylarginine; SDMA, Symmetric dimethylarginine; SM (OH): sphingolipid; lysoPC: lysophosphatidylcholine; PC: phosphatidylcholine.

Table S6. Summary of the NMR results. Metabolites in plasma important for separation of the PD, MSA, and PSP patient groups from the control group, and from each other, in the multivariate statistical models generated from the NMR data. Increased and decreased concentrations of the metabolites are indicated with an upward or downward arrow, respectively. Bold arrows indicate metabolites with a *p*-value below 0.05 in the univariate analysis.

Metabolite	*PD*	*MSA*	*PSP*	*PD*	*PD*	*MSA*
		vs			*vs*	
		Controls		*MSA*	*PSP*	*PSP*
Formic acid	⬇	⬇	⬇		⬆	⬆
Succinate	⬆	⬆	⬆			
L-Arginine	⬆		⬆	⬆		⬇
Proline	↑					
Creatinine	↑					
L-Lysine	⬇				⬇	
3-HIB	↑			↑		
Glutamate	↑					
L-Histidine	↑				⬆	↑
Taurine		⬇	⬇	⬆	⬆	
Acetoacetate		↓		↑		↓
L-Glutamine		↑				↑
Lactic acid		⬆				
TMAO		↑				
2-HB		↓		⬆		
3-HB		↓		↑		
Dimethylamine			⬇		⬆	↑
Pyruvic acid			⬆			
L-Isoleucine				↑	⬆	
L-Leucine				↑	⬆	
L-Valine				↑	↑	
L-Tyrosine					↑	
L-Methionine					↑	

TMAO: Trimethylamine N-oxide; HB: hydroxybutyrate;
HIB: hydroxyisobutyrate

Table S7. Summary of the MS results. Metabolites in plasma important for the separation of the PD, MSA, and PSP patient groups from the control group, and from each other, in the multivariate statistical models generated from the MS data. Increased and decreased concentrations of the metabolites are indicated with an upward or downward arrow, respectively. Bold arrows indicate metabolites with a *p*-value below 0.05 in the univariate analysis.

Metabolite	*PD*	*MSA* *vs* *Controls*	*PSP*	*PD* *MSA*	*PD* *vs* *PSP*	*MSA* *PSP*
PC aa C36:0	⬇	⬇	⬇	↑		
PC aa C38:1	⬇	⬇				
PC ae C40:2	⬇	⬇				
lysoPC a C24:0	⬇	⬇				
lysoPC a C26:0	⬇	⬇			⬇	⬇
lysoPC a C26:1	⬇	⬇		↑		⬇
PC ae C36:0	⬇		⬇			
carnitine	⬆		⬆	⬆	⬇	⬇
propionylcarnitine	⬆					
lysoPC a C28:1	⬇					
PC ae C30:2	⬇					
PC ae C36:3	⬇					
PC ae C40:3	⬇					
PC ae C42:3	⬇					
Sarcosine		⬇	⬇		⬆	⬆
PC aa C42:2		⬇				
lysoPC a C18:0		⬇				
SM (OH) C22:1		⬇				
acetylcarnitine			⬆	⬆		⬇
Serotonin			⬆			
PC ae C44 :5			⬆			
Glycine				⬇		
Creatinine				⬆		
Kynurenine				⬆		
L-Isoleucine				⬆	↑	
L-Leucine				⬆		
L-Valine				⬆	⬆	
L-Glutamine				⬆		↓
L-Histidine					⬆	↑
L-Tyrosine					↑	
L-Tryptophan					↑	
lysoPC a C28:0					⬇	⬇
PC aa C36:3					⬇	
PC aa C40:3						↓
C14:1						⬇

C14:1, tetradecenoylcarnitine; SM (OH): sphingolipid; lysoPC: lysophosphatidylcholine; PC: phosphatidylcholine.
